# Vitamin D and rosuvastatin alleviate type-II diabetes-induced cognitive dysfunction by modulating neuroinflammation and canonical/noncanonical Wnt/β-catenin signaling

**DOI:** 10.1371/journal.pone.0277457

**Published:** 2022-11-14

**Authors:** Muhammad Muneeb, Suzan M. Mansou, Samira Saleh, Reham A. Mohammed

**Affiliations:** 1 Department of Pharmacology, Toxicology, and Biochemistry, Faculty of Pharmacy, Future University in Egypt, Cairo, Egypt; 2 Department of Pharmacology and Toxicology, Faculty of Pharmacy, Cairo University, Cairo, Egypt; Universidade do Estado do Rio de Janeiro, BRAZIL

## Abstract

**Background:**

Type-II diabetes mellitus (T2DM) is a major risk factor for cognitive impairment. Protecting the brain environment against inflammation, and neurodegeneration, as well as preservation of the BBB veracity through modulating the crosstalk between insulin/AKT/GSK-3β and Wnt/β-catenin signaling, might introduce novel therapeutic targets.

**Purpose:**

This study aimed at exploring the possible neuroprotective potential of vitamin D3 (VitD) and/or rosuvastatin (RSV) in T2DM-induced cognitive deficits.

**Methods:**

T2DM was induced by a high-fat sucrose diet and a single streptozotocin (STZ) dose. Diabetic rats were allocated into a diabetic control and three groups treated with RSV (15 mg/kg/day, PO), VitD (500 IU/kg/day, PO), or their combination.

**Results:**

Administration of VitD and/or RSV mitigated T2DM-induced metabolic abnormalities and restored the balance between the anti-inflammatory, IL 27 and the proinflammatory, IL 23 levels in the hippocampus. In addition, they markedly activated both the canonical and noncanonical Wnt/β-catenin cassettes with stimulation of their downstream molecular targets. VitD and/or RSV upregulated insulin and α7 nicotinic acetylcholine (α7nACh) receptors gene expression, as well as blood-brain barrier integrity markers including Annexin A1, claudin 3, and VE-cadherin. Also, they obliterated hippocampal ApoE-4 content, Tau hyperphosphorylation, and Aβ deposition. These biochemical changes were reflected as improved behavioral performance in Morris water maze and novel object recognition tests and restored hippocampal histological profile.

**Conclusion:**

The current findings have accentuated the neuroprotective potential of VitD and RSV and provide new incentives to expand their use in T2DM-induced cognitive and memory decline. This study also suggests a superior benefit of combining both treatments over either drug alone.

## 1. Introduction

Type-II diabetes mellitus (T2DM) is a major risk factor for cognitive impairment [1–3]. Insulin receptors are widely distributed in the brain [4, 5] with similar kinetics and pharmacological properties to those present in peripheral tissues [6–8] and ultimately insulin plays a critical role in modulating cognitive performance [9, 10].

At the molecular level, impaired insulin signaling may promote amyloid-β (Aβ) deposition and Tau hyperphosphorylation via brain insulin resistance, which disturbs insulin signaling at the blood-brain barrier (BBB) level [11, 12] through the Wingless-related integration site (Wnt)/glycogen synthase kinase-3 β (GSK-3β)/β-catenin signaling pathway. This leads to neuronal death and behavioral deficits possibly by promoting β-catenin degradation [13]. Studies have shown that both canonical and noncanonical Wnt/β-catenin pathways play a significant role in learning and memory [14, 15], as well as synaptic plasticity and cell survival [13].

The canonical pathway is activated when the Wnt-5a ligand binds to its receptor thus phosphorylating β-catenin at serine (S) 675. As a consequence, β-catenin accumulates in the cytosol and subsequently translocates to the nucleus where it promotes Wnt target genes expression [14]. Conversely, studies have shown that GSK-3β activation promotes β-catenin phosphorylation at S37 in the absence of Wnt ligands thus facilitating β-catenin degradation [16, 17]. Hence, contributes to neuronal pathology, and cognitive and memory shortage [15]. In the non-canonical Wnt pathway, activation of homolog family member A (RhoA) and rac family small GTPase 1 (Rac1) increase the phosphorylation of (protein kinase-B) AKT and subsequently GSK-3β [18]. This phosphorylation process decreases Aβ aggregation, and Tau deposition and leads to translocation of β-catenin into the nucleus, and consequently improves cognitive deficits [17].

Emerging evidence also suggests that blood-brain barrier (BBB) integrity is crucial in the pathology of neurodegeneration and cognitive impairment. BBB disruption resulting from multiple neuroinflammatory events that interrupt tight junctions is a marked feature of cognitive defects [19]. Thus, protecting the brain environment against inflammation, and neurodegeneration, as well as preservation of the BBB veracity through modulating Wnt/β-catenin signaling, might introduce novel therapeutic targets for T2DM-associated cognitive decline.

Rosuvastatin (RSV) is an HMG-CoA reductase inhibitor used in the management of dyslipidemia [20]. Lowering cholesterol levels in experimental animal models has been proven to slow down the progression of learning and memory deficits [21]. Regarding the role of statins in both cognitive impairment and protection against dementia, data in the literature are contradictory, ranging from the evidence of a reversible cognitive impairing effect in some patients to a protective effect; some authors do not suggest an effect of statins on cognition [22–25]. The widespread use of statins heightens the importance of careful consideration of this effect. Moreover, it has been reported that statins could reduce the risk of dementia and cognitive decline directly by promoting the Wnt/β-catenin signaling pathway [13, 26]. Accordingly, further studies are required to characterize the intracellular signaling transduction that derives its protective effect against cognitive deterioration in T2DM.

Vitamin D_3_ (VitD), a well-known secosteroid hormone, exerts both genomic and non-genomic actions; these actions cooperate by crosstalk between several signaling pathways. It has been increasingly implicated in the pathophysiology and the progression of many neurological diseases [27] including Alzheimer’s disease (AD) [28] and ischemic stroke [29]. Current evidence suggests that VitD may be an interesting candidate for T2DM pathogenesis and development [30] and that it could maintain cognitive function because of its neuroprotective, anti-inflammatory, and antioxidant properties [31, 32]. In the brain, VitD was shown to affect neurite growth, differentiation, synaptic plasticity, as well as neuroprotection [31, 33, 34]. However, the possible therapeutic contribution of VitD in cognitive disorders in T2DM is still questioned.

To this end, the present study aims at investigating the possible benefits of VitD and/or RSV in rats in T2DM-induced cognitive and memory loss. Additionally, this work addresses the potential modulatory role of the crosstalk between insulin and Wnt/β-Catenin cassettes, and their downstream targets in the observed beneficial outcomes.

## 2. Materials and methods

### 2.1. Animals

Adult male Sprague Dawley rats (150–180 g) were purchased from the breeding colony of the National Institute of Research (Giza, Egypt). Rats were kept under standardized laboratory conditions with food and water *ad libitum*. They were exposed for 12 h light/dark cycle and controlled temperature (25±5֯C). The study protocol was approved by the Research Ethics Committee of the Faculty of Pharmacy, Cairo University, Cairo, Egypt (PT-2310) and the Faculty of Pharmacy (Future University in Egypt, Cairo, Egypt) along the lines of the Guide for the Care and Use of Laboratory Animals (ILAR, 2001) [35].

### 2.2. Drugs and chemicals

Streptozotocin (STZ) and RSV were purchased from Sigma-Aldrich Co., St. Louis, MO, USA; VitD was obtained from Medical Union Pharmaceuticals Co., Cairo, Egypt; cholesterol and long-acting human insulin (Monotard) were obtained from Middle East Co., Cairo, Egypt, and Eli Lilly Co., USA, respectively. Sucrose and lard were obtained from commercial sources and were of the highest analytical grade.

### 2.3. Induction of T2DM-induced cognitive impairment and experimental design

Forty rats (approximately 5–6 weeks in age) were fed a high-fat sucrose diet (HFSD) for 11 weeks, according to the method of *Cai et al* [36], with slight modification. The diet was composed of 20% sucrose, 25% lard, 2.5% cholesterol, and 52.5% standard chow [composed of fat (5%), protein (26%), carbohydrate as starch (60%), fibers (8%), and vitamins/minerals mixture (1%)]. At the beginning of the 5^th^ week, a single sub-diabetogenic dose of STZ (35 mg/kg; IP) dissolved in 0.09 M citrate buffer solution (pH 4.8), was given after an overnight fast. Animals were then maintained on a 5% glucose solution for 24 h. A normal-control (NC; n = 10) group was kept on a conventional pellet diet and water *ad libitum* was run concomitantly. The T2DM model was considered successful when the random blood sugar level was above 200 mg/dl at the beginning of the 7^th^ week [36].

After establishing the model (week 7), HFSD-fed animals were randomly allocated into four groups (10 rats/ each); T2DM, T2DM + VitD, T2DM + RSV, and T2DM + VitD + RSV. Then, the rats were treated daily for 5 weeks (weeks 7–11) with the drugs along with HFSD. The dose of VitD was 500 IU/kg/day; PO [37], while that for RSV was 15mg/kg/day; PO [38].

### 2.4. Behavioral studies

At the beginning of the 11^th^ week, all animals were subjected to the novel object recognition and Morris water maze tests to assess learning ability, and cognitive and memory impairment.

#### 2.4.1. Novel Object Recognition Test (NORT)

NORT is used to assess long-term memory and cognition [39]. It consists of habituation, familiarization, and test sessions. In habituation, animals were placed in a wooden box of 30 × 70 × 70 cm dimensions and allowed to discover it for 10 min for two consequent days. On the third day, each rat was placed in the same apparatus, which contained two identical objects (A + A) placed side by side, for 10 min (familiarization). Twenty-four hours thereafter, animals were subjected to the testing session where one of the previously explored objects was replaced by a novel one (A + B). Animals were then put back in the middle of the box with two objects (A + B) for 10 min. The objects used in this experimentation were mostly small toys (8–12 cm) with a variety of textures, structures, colors, and sizes, which were fixed on the floor with removable adhesive tape with their edges at 15 cm from the walls. Rats’ behavior during the test was recorded using a camera [40]. For each animal, the percentage of time spent exploring the novel object (novel object/[novel object + old object] ×100) and the old object (the % of novel object—100) during the test session was calculated [39]. A discrimination index was determined using this formula (novel object − old object)/ (novel object + old object) [40].

#### 2.4.2. Morris Water Maze Test (MWMT)

MWMT assesses spatial learning [41]. It is a large open circular pool (160 cm in diameter, 50 cm in height) half-filled with water at a temperature of 22°C ± 1. The water surface was divided into four quadrants. To render the platform invisible, non-toxic white latex paint was added and a white escape platform (11 cm in diameter) was submerged 1 cm beneath the water level. The procedure was performed on five consecutive days. Rats were submitted to four trials each day and started from randomly set positions. In each trial, rats were allowed to swim for 120 s. If the rat was unable to locate the platform during this period, it was guided to the platform and left for 30 s. The platform was always in the same position during all training trials. The mean escape latency (MEL) to reach the platform, and the time spent in the target quadrant was measured on day 5 whereby the platform was removed [40].

### 2.5. Collection of blood samples

After the last dose of the drugs, animals were fasted for 12 h, anesthetized with thiopental (60 mg/kg, IP) and blood samples were collected from the heart following chest opening. Serum was separated by centrifugation at 3000 rpm for the estimation of glucose, total cholesterol (TC), triglycerides (TGs), and high-density lipoprotein cholesterol (HDL-C) using colorimetric assay kits (SPECTRUM^®^, Egypt). Low-density lipoprotein cholesterol (LDL-C) was calculated according to the Friedewald equation: TC−(HDL cholesterol+1/5 TGs) [42]. Free fatty acids (FFAs) and insulin were measured by ELISA kits (MyBioSource^®^, USA) and (RayBiotech^®^, GA, USA; #ELR-Insulin), respectively. Homeostasis model assessment for insulin resistance (HOMA-IR) was estimated according to the following equation:

$$\text{HOMA-IR}=\text{Glucose}\;\left(\text{mg}/\text{dl}\right)\times \text{fasting}\;\text{insulin}\;\left(\text{mIU}/\text{ml}\right)/405$$

[43].

### 2.6. Tissue Preparation and biochemical investigations

Following the collection of blood samples, brains (n = 4) were dissected and preserved in 10% formalin in saline for histopathological and immunohistochemical studies. Hippocampi from the remaining rats (n = 6) were excised and stored at -80°C. The left hippocampus was homogenized in ice-cold saline to prepare 10% homogenate to be assayed using the ELISA technique. While the right hippocampi were divided into two subsets. One subset (3 rats) was homogenized in a radioimmunoprecipitation assay (RIPA) buffer with protease and phosphatase inhibitors and was divided into aliquots for Western blotting analysis and the other one (3 rats) was submerged overnight in RNA lysis solution for the qRT-PCR assay.

The Bradford assay was used for the estimation of the protein content of the homogenized samples [44].

#### 2.6.1. ELISA technique

Interleukin-23 (IL-23), interleukin-27 (IL-27), apolipoprotein E type-4 allele (ApoE-4), claudin-3 and vascular endothelial cadherin (VE-cadherin) contents were determined using ELISA kits (MyBioSource^®^, USA); with catalog numbers: MBS704680, CSB-E08465r, MBS263133, MBS451608 and MBS2703236, respectively. All procedures were performed according to the manufacturers’ instructions. The results are presented as ng/mg protein for ApoE-4, VE-cadherin and pg/mg protein for Claudin-3, IL-23, IL-27.

#### 2.6.2. Western blot analysis

Following protein quantification of hippocampal tissue (Bio-Rad Protein Assay Kit, CA, USA), protein extracts were separated by SDS gel electrophoresis and then transferred to nitrocellulose membrane. The blots were probed with antibodies (ThermoFisher Scientific, MA, USA) specific for Wnt-5a (1:1000; cat#: MA5-14946), *p*-tau (Ser396; 1:1000, #44-752G), *p*GSK-3β (Ser9; 1:1000, cat#: MA5-14873), *p*-AKT (Ser473; 1:500–1:3000, cat#: PA5-85513), RhoA (1:500–1:2000; cat#: MA1-134), Rac1 (1:500–1:1000; cat#: MA5-32928), *p*S675 β–catenin (1:1000–1:3000; cat#: PA5-105840) and *p*S37 β–catenin (1:500–1:2000; cat#:PA5-104871) Horseradish peroxidase-conjugated goat anti-rat immunoglobulin (Dianova, Hamburg, Germany) was used as the secondary antibody, which is a Horseradish peroxidase-conjugate (cat#: NBP1-75304). Immunoreactivity was detected by CCD camera-based imager and band intensities of the target proteins were normalized against the control sample (β-actin) (cat#: MA1115) using Chemi Doc MP Imager. Results are expressed as arbitrary units against β-actin.

#### 2.6.3. Quantitative RT-PCR technique

Total RNA was extracted from hippocampal sections using SV total RNA isolation system (Promega, Madison, WI, USA) and the purity of RNA was verified at 260 nm by spectrophotometer. The extracted RNA was conversely transcribed into cDNA using RT-PCR kit (Stratagene, Santa Clara, CA) according to the manufacturer’s guidelines. Gene expression levels were assessed by SYBR Green-based Real Time Quantitative PCR method. Table 1 demonstrate PCR primers designed with Gene Runner Software (Hasting Software, Inc., Hasting, New York) from RNA sequences from GenBank. All primer sets had a calculated annealing temperature of 60°C. Amplification conditions were 2 minutes at 50°C, 10 minutes at 95°C and 40 cycles of denaturation for 15 seconds and annealing/extension at 60°C for 10 minutes. For quantification of mRNA, comparative Ct method (ΔCt value) was used, where the quantity of target transcript was normalized according to the level of beta actin gene using StepOne Applied Biosystems Software (Foster City).

**Table 1:** Primer sequences for quantitative PCR of the studied genes.

Studied gene	Primer sequence	Gene bank accession number
Insulin receptor	Forward: 5′-TTCATTCAGGAAGACCTTCGA-3′	XM_039089098.1
	Reverse: 5′-AGGCCAGAGATGACAAGTGAC-3′	
α7nAch receptor	Forward: 5′CTGGTGCCAGCAGTGTTGAC3’	NM 133420.1
	Reverse: 5′GATTGTAGCCTCCAAACAGGTGT3	
Annexin A1	Forward: 5′- GCCCCTACCCTTCCTTCAAT-3′	NM_012904.2
	Reverse: 5′- GAGTGTCTTCATCTGTTCCA-3′	
β-actin	Forward: 5′-AGGCATCCTCACCCTGAAGTA-3′	NM_031144.3
	Reverse: 5′-CACACGCAGCTCATTGTAGA-3′	

#### 2.6.4. Histopathological and immunohistochemical investigations

Hippocampi were fixed in 10% phosphate-buffered formalin for 72 h. Tissue specimens were embedded in paraffin wax and sectioned at 5 μm thickness and stained with hematoxylin and eosin. Stained sections were blindly examined under a light electric microscope (Olympus CX21, Tokyo, Japan) and photographed with a CCD camera-based imager. Coronally cut sections (4 μm) were also prepared for immunohistochemical staining of Amyloid-β (Aβ) using polyclonal Aβ antibody 4702 (1:1500) and monoclonal Aβ antibodies 6E10 (1:2000–4000; Senetek, Maryland Heights, MO) and 4G8 (1:20,000; Senetek). Diaminobenzidine was used for staining plaque-associated immunoreactivity. The severity of the injury was semi-quantitatively scored as 0 (no staining), 1+ (<10 plaques), 2+ (>10 scattered plaques), 3+ (most of the hippocampus stained), or 4+ (almost confluent staining).

### 2.7. Statistical analysis

Data are expressed as means ± SD. For parametric analysis, multiple comparisons were performed using a one-way analysis of variance (ANOVA) test followed by Tukey’s Multiple Comparison Test. For non-parametric data, Kruskal–Wallis followed by Dunn’s multiple comparisons tests was used. GraphPad Prism software package, version 7 (GraphPad Software Inc., CA, USA) was used to carry out all statistical tests. The level of significance was fixed at *p* < 0.05 for all statistical tests.

## 3. Results

### 3.1. VitD, RSV, and their combination improved T2DM-induced cognitive impairment

**Fig 1:**
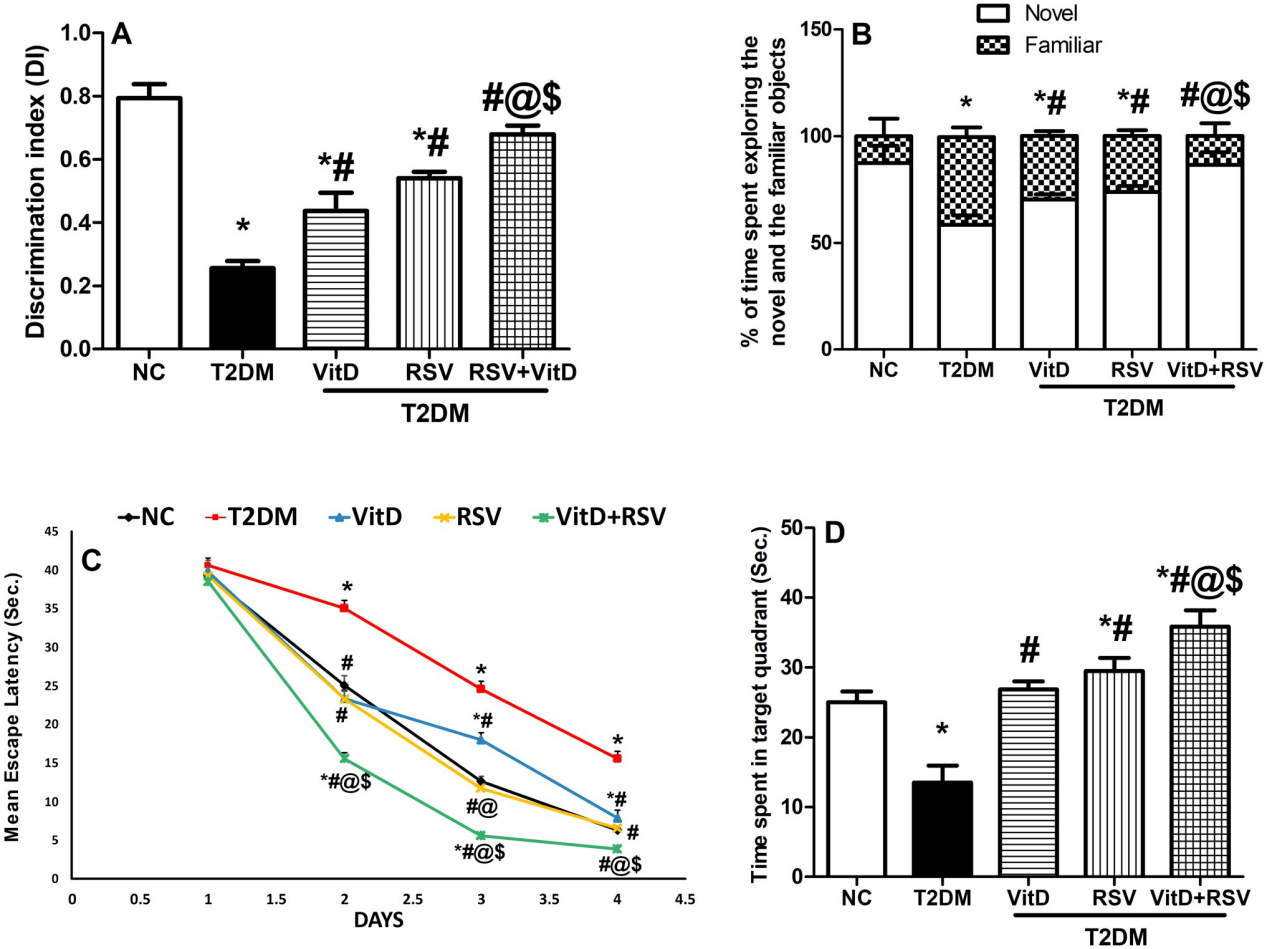
Effect of VitD and/or RSV on T2DM-induced cognitive impairment in the NORT and the MWMT. (A) discrimination index (NORT); (B) a percentage of time spent exploring the novel and the familiar objects (NORT); (C) mean escape latency (MWMT); (D) time spent in the target quadrant (MWMT). Data are represented as mean ± SD (n = 10). * vs control, ^#^ vs T2DM, ^@^ vs VitD, ^$^ vs RSV using one-way ANOVA followed by Tukey multiple comparison test at *p*<0.05. NC: normal-control, RSV: rosuvastatin, T2DM: type-II diabetes mellitus, VitD: vitamin D_3_.

As revealed in Fig 1, diabetic rats showed marked cognitive deficits in both NORT and MWM tests. In the NORT, diseased rats showed a 32% decrement in the discrimination index **(A)** and 67% in the percentage of time spent exploring the new object **(B)**, indicating long-term memory deterioration. Treatment with either VitD or RSV improved the discrimination index and shortened the time spent exploring the familiar object compared to the T2DM group. The combination group significantly restored the abovementioned parameters to near normal values.

In the MWMT, the mean escape latency (MEL) was increased by 2.5 folds compared to the NC group **(C)**. Additionally, in the probe test, T2DM rats spent 54% less time in the target quadrant **(D)** searching for the missing platform. Treatment with VitD, RSV, and their combination significantly decreased the mean escape latency, and the time spent in the target quadrant compared to T2DM, indicating improved spatial learning and memory tasks.

### 3.2. VitD, RSV, and their combination improved T2DM-induced histopathological alterations and Aβ deposition

**Fig 2:**
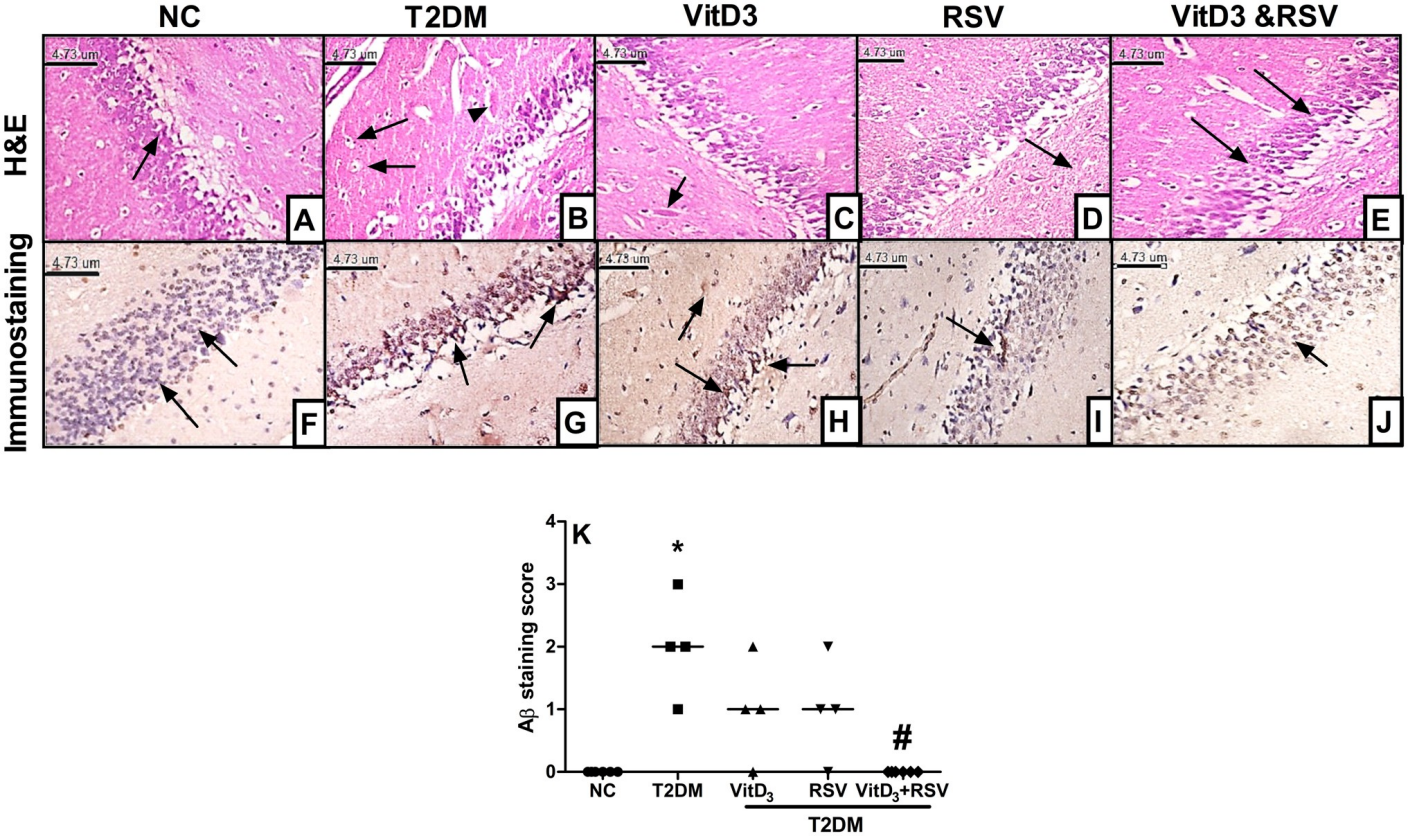
Effect of VitD and/or RSV on T2DM-induced histological changes. A-E Specimens stained with H&E (400 x); (**A)** Control group showing normal histological structure with normal granular cell layers *(arrow)*, (**B)** T2DM group with relatively few numbers of neurofibrillary tangles and Aβ formation appeared as flame-shaped structures *(arrowhead)* and rod-shaped, crystal-like, eosinophilic intra-neural structures known as Hirano bodies *(arrow)*, (**C)** VitD group showing Aβ formation *(arrow)*, (**D)** RSV group representing few numbers of faint Aβ *(arrow)* and (**E)** combination group demonstrating the normal structure of glial and pyramidal cells *(arrow)*. *F-J* immunostaining of the hippocampal area (400 x); (**F)** Control group showing no expression of Aβ *(arrow)*, (**G)** T2DM group revealing a huge expression of Aβ (>10 stained scattered plaques) *(arrow)*, **(H)** VitD group showing a moderate expression of Aβ (<10 stained scattered plaques) *(arrow)*, (**I)** RSV group representing a slight expression of Aβ (<10 stained plaques) *(arrow)*, and (**J)** combination group showing no expression of Aβ *(arrow*). (**K)** Aβ staining score. Data are represented as a scattering dotted plot of the median of 4 sections of 4 animals. * vs control, ^#^ vs T2DM. Statistical analysis was performed by Kruskal-Wallis followed by Dunn’s multiple comparison test at p<0.05. Aβ: amyloid-β, NC: normal-control, RSV: rosuvastatin, T2DM: type-II diabetes mellitus, VitD: vitamin D_3_.

As shown in Fig 2, moderate neurofibrillary tangles and Aβ formation were exhibited in the diabetic group **(B)** compared to the NC group **(A)**. Hirano bodies which are a main feature of neurodegeneration were also detected. Additionally, immunostaining of the hippocampal area **(F-J)** revealed a huge deposition of Aβ (>10-stained scattered plaques; **(G)** compared to normal rats **(F)**. The VitD-treated group displayed fewer Aβ plaques **(H)** with a moderate Aβ expression (<10 stained scattered plaques; H), while in the RSV-treated group, there were only a few numbers of plaques **(I)** with faint staining of Aβ (<10 stained plaques; I). Interestingly, the combined treatment **(E)** showed the normal structure of glial cells and pyramidal cells with no expression of Aβ **(J)**. The observed Aβ staining score is portrayed in panel **K**.

### 3.3. VitD, RSV, and their combination improved T2DM-induced metabolic dysfunction

**Fig 3:**
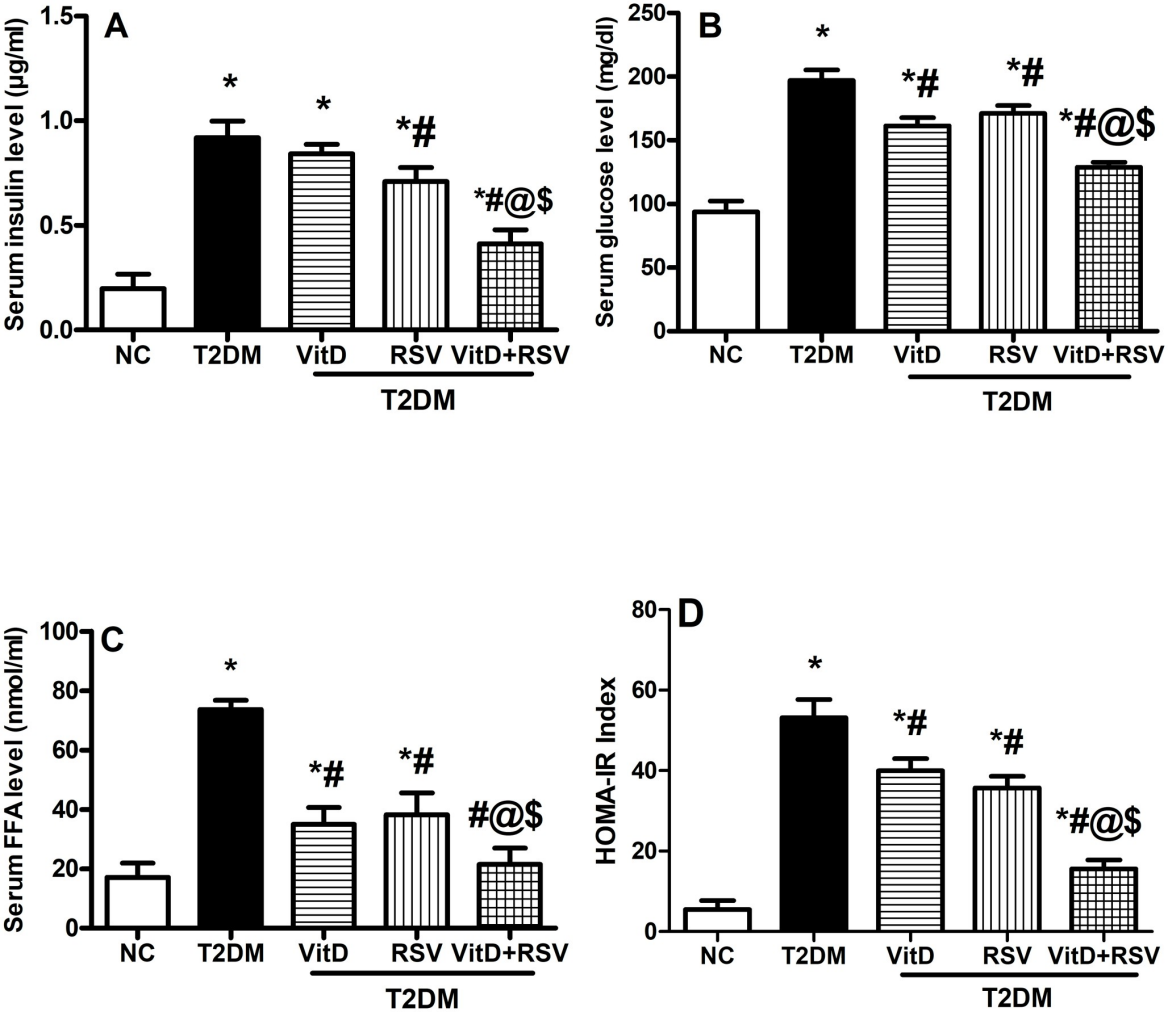
Effect of VitD and/or RSV on T2DM-induced metabolic disturbance in the serum. (A) Insulin, (B) Glucose, and (C) FFAs levels, as well as (D) The pattern of the HOMA-IR index. Data are expressed as mean ± SD. * vs control, ^#^ vs T2DM, ^@^ vs VitD, ^$^ vs RSV using one-way ANOVA followed by Tukey multiple comparison test at p<0.05. FFAs: free fatty acids, HOMA-IR: homeostasis model assessment for insulin resistance, NC: normal-control, RSV: rosuvastatin, T2DM: type-II diabetes mellitus, VitD: vitamin D_3_.

As cleared in Fig 3, diabetic rats showed a threefold elevation in the level of serum insulin **(A)**, a twofold rise in serum glucose level **(B)**, and a threefold increase in free fatty acids **(C)** as compared to the NC group. Conversely, administration of either VitD or RSV resulted in a significant reduction in glucose and FFAs levels, compared to T2DM. However, serum insulin level was significantly reduced by RSV treatment only. In the combination treatment, a more pronounced attenuation of the abovementioned parameters was reached as compared to either drug alone. HOMA-IR values in diabetic rats were drastically elevated to 9.7 times the NC group **(D)**, while combined VitD and RSV therapy changed it to 3.4 times.

### 3.4. VitD, RSV, and their combination improved T2DM-accompanied dyslipidemia

**Fig 4:**
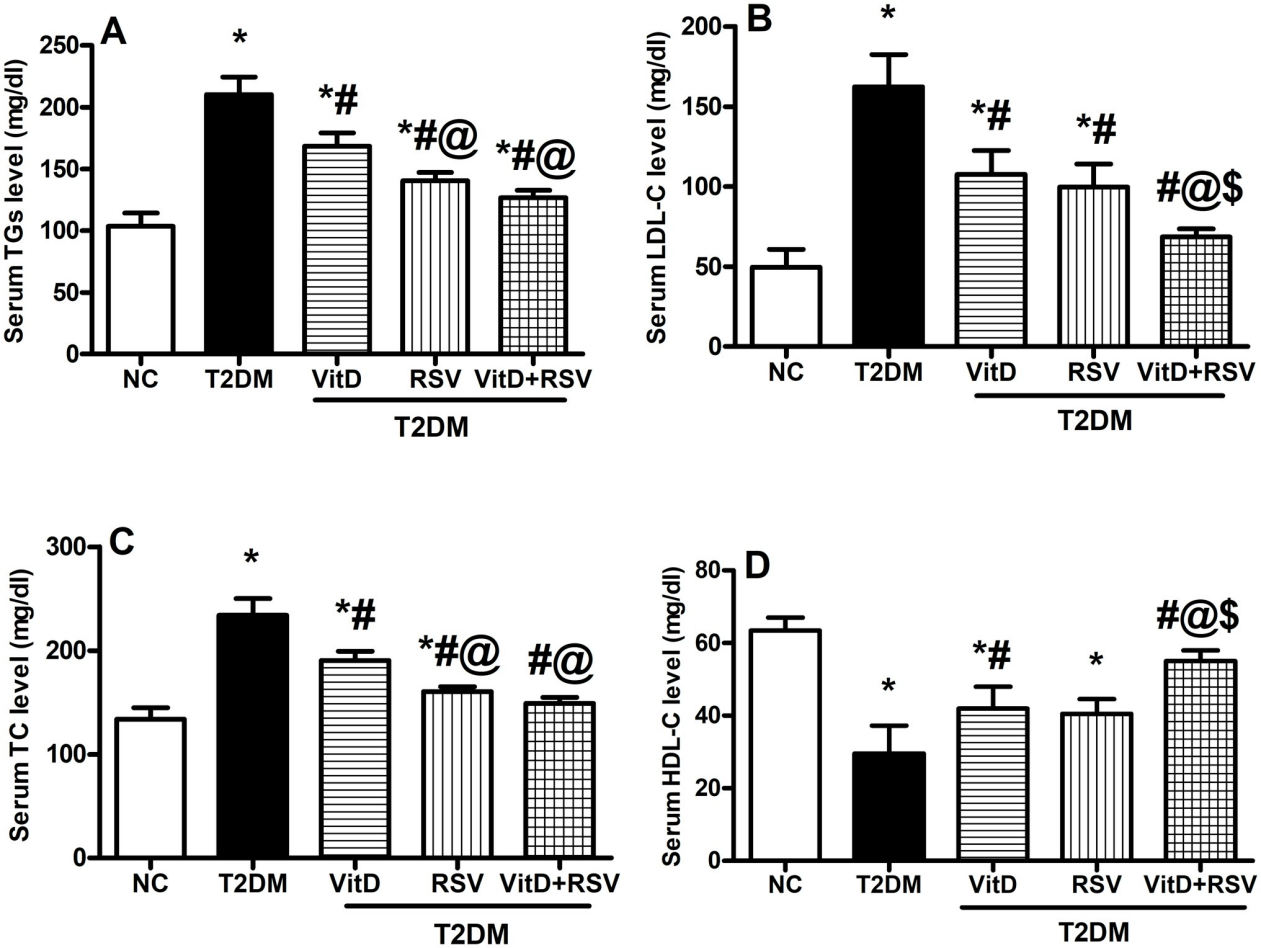
Effect of VitD and/or RSV on T2DM-induced disturbance in lipid profile: (A) TGs, (B) LDL-C, (C) TC, and (D) HDL- C levels. Data are expressed as mean ± SD. * vs control, ^#^ vs T2DM, ^@^ vs VitD, ^$^ vs RSV using one-way ANOVA followed by Tukey multiple comparison test at p<0.05. HDL-C: high-density lipoprotein cholesterol, LDL-C: low-density lipoprotein-density cholesterol, NC: normal-control, RSV: rosuvastatin, TC: total cholesterol, TGs: triglycerides, T2DM: type-II diabetes mellitus, VitD: vitamin D_3_.

Fig 4 showed that diabetic rats demonstrated an obvious twofold elevation in the serum levels of TGs **(A)**, threefold elevation in LDL-C **(B)**, and a twofold increase in TC **(C)** accompanied by a marked 53.5% reduction in HDL-C **(D)** in comparison to the NC group. Notably, treatment with VitD significantly decreased TGs, LDL-C, and TC together with a profound boost in HDL-C levels. In parallel, administration of RSV markedly reduced TGs, LDL-C, and TC levels, but failed to raise the level of HDL-C to any significant extent. Again, combined treatment with VitD and RSV resulted in a more favorable effect on the previous parameters.

### 3.5. VitD, RSV, and their combination improved hippocampal T2DM-induced alterations in the canonical Wnt/β-catenin signaling pathway

**Fig 5:**
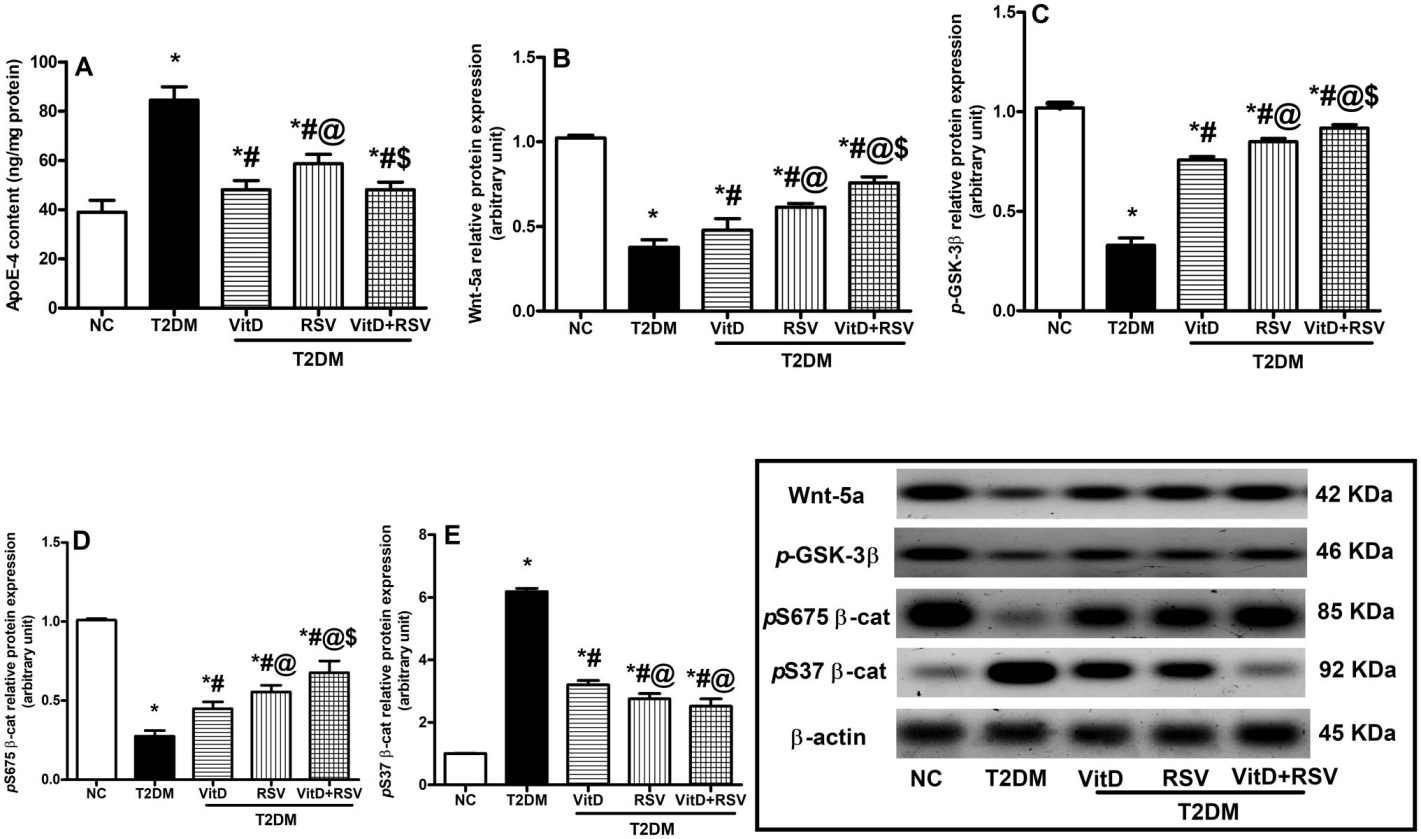
Effect of VitD and/or RSV on the canonical Wnt/β-catenin signaling pathway. (A) ApoE-4 content, protein expression of (B) Wnt5a, (C) *p*S9GSK-3β, (D) *p*S675 β-catenin and (E) *p*S37 β-catenin. Data are expressed as mean ± SD. * vs control, ^#^ vs T2DM, ^@^ vs VitD, ^$^ vs RSV using one-way ANOVA followed by Tukey multiple comparison test at p<0.05. ApoE-4: apolipoprotein E type 4 allele, *p*GSK-3β: phosphorylated glycogen synthase kinase-3 β, NC: normal-control, RSV: rosuvastatin, T2DM: type-II diabetes mellitus, VitD: vitamin D_3_, Wnt5a: wingless-type family member 5a.

As expressed in Fig 5, T2DM markedly inactivated the canonical Wnt/β-catenin signaling pathway as indicated by a twofold rise in ApoE-4 content **(A)**, a fall of nearly 63% of Wnt5a **(B)**, 68% of *p*S9 GSK-3β **(C)** and a 73% of *p*S675 β-catenin **(D)**. This was paralleled by a six-fold upregulation of *p*S37 β-catenin **(E)** as compared to the NC group. Treatment with either VitD or RSV significantly decreased ApoE-4 content and upregulated Wnt5a, *p*S9GSK-3β, and *p*S675 β-catenin together with a downregulation in *p*S37 β-catenin as compared to T2DM group. Notably, the combined therapy showed more prominent improvement over either drug alone.

### 3.6. VitD, RSV, and their combination attenuated T2DM-induced inhibition of the non-canonical Wnt/β-catenin signaling pathway

**Fig 6:**
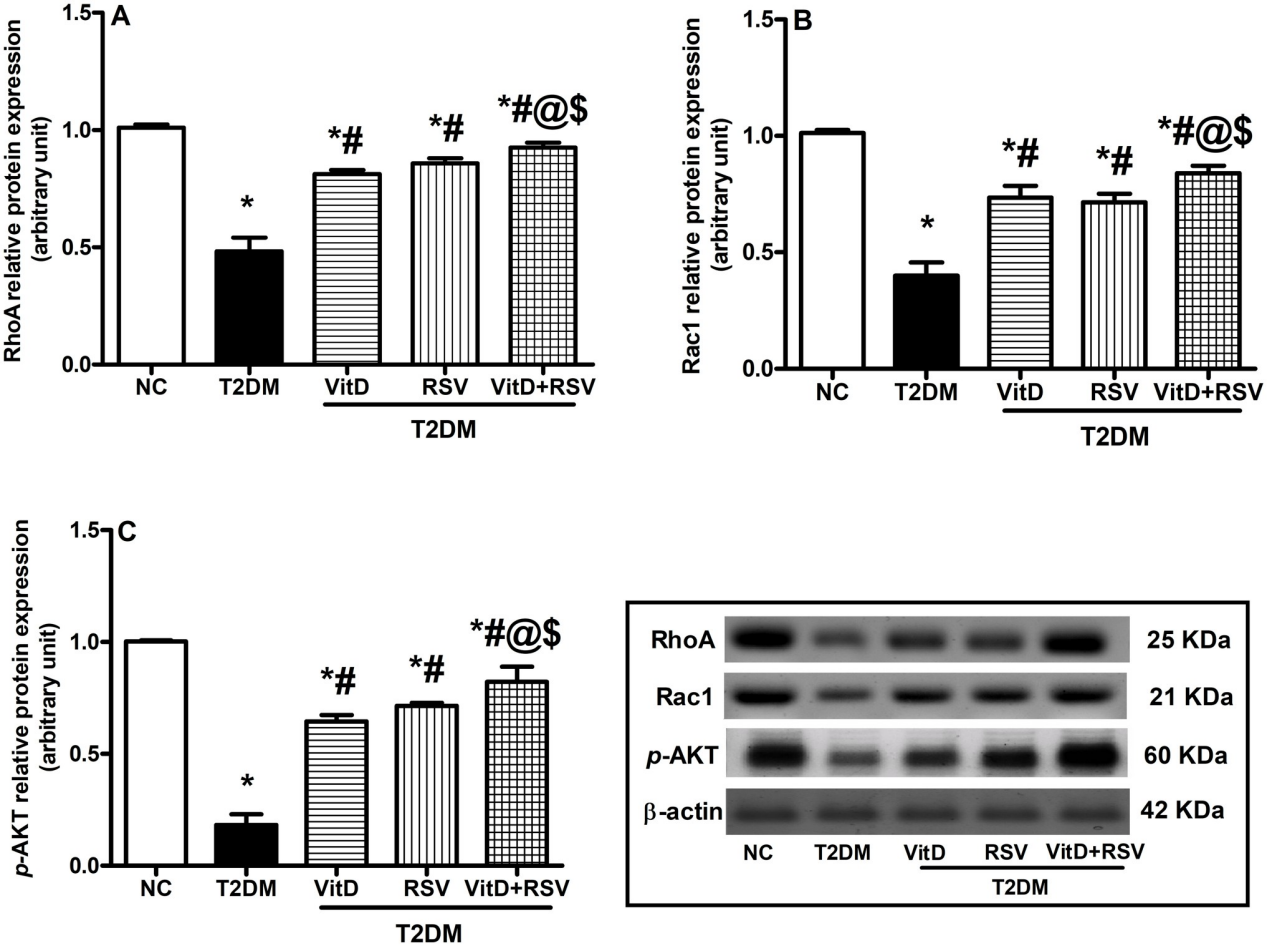
Effect of VitD and/or RSV on the noncanonical Wnt/β-catenin signaling pathway. (A) RhoA; (B) Rac1 and (C) *p-*AKT protein expression. Data expressed as mean ± SD. * vs control, ^#^ vs T2DM, ^@^ vs VitD, ^$^ vs RSV using one-way ANOVA followed by Tukey multiple comparison test at p<0.05. NC: normal-control, *p*-Akt: phosphorylated-protein kinase-B, Rac1: rac family small GTPase 1, RhoA: ras homolog family member A, RSV: rosuvastatin, T2DM: type-II diabetes mellitus, VitD: vitamin D_3_.

Diabetic rats presented an obvious 52.3% and 60.5% reduction in RhoA (Fig 6A) and Rac1 (Fig 6B) relative protein expression, respectively as compared to the NC group. Additionally, a significant 81.8% decrease in the phosphorylation of Akt at S473 was observed (Fig 6C). In comparison with the T2DM group, treatment with either VitD and/or RSV significantly reversed the previous effects.

### 3.7. VitD, RSV, and their combination increased hippocampal claudin 3, VE-cadherin contents, and Annexin A1 gene expression

**Fig 7:**
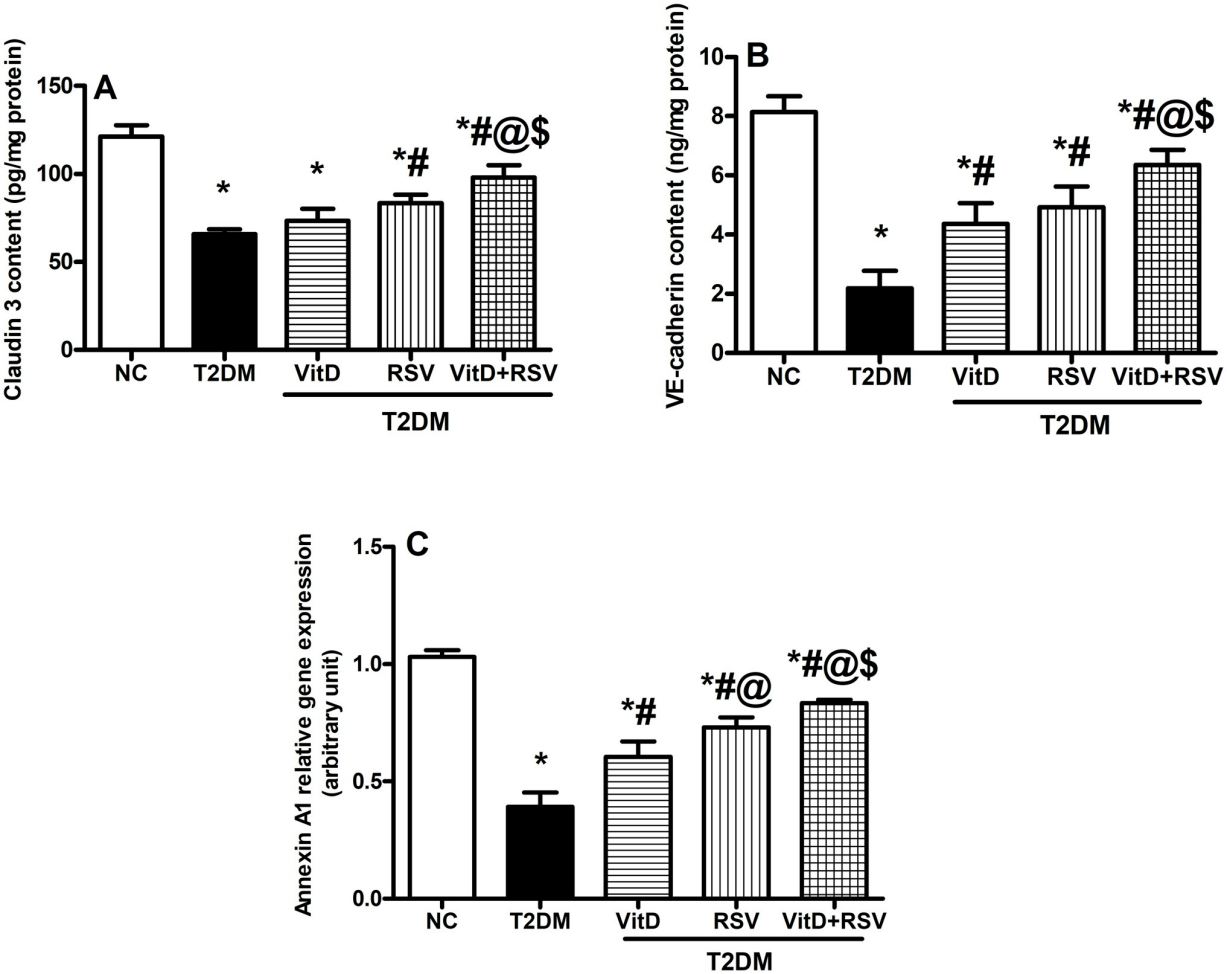
Effect of VitD and/or RSV on the indicators of BBB integrity. (A) claudin-3, (B) VE-cadherin contents, and (C) Annexin A1 relative gene expression. Data are expressed as mean ± SD. * vs control, ^#^ vs T2DM, ^@^ vs VitD, ^$^ vs RSV using one-way ANOVA followed by Tukey multiple comparison test at p<0.05. NC: normal-control, RSV: rosuvastatin, T2DM: type-II diabetes mellitus, VE-cadherin: vascular endothelial-cadherin, VitD: vitamin D_3_.

T2DM rats manifested a 45.7%, 73%, and 69.8% decline in the hippocampal claudin 3 (Fig 7A), VE-cadherin (Fig 7B) contents, and Annexin A1 relative gene expression (Fig 7C), respectively as compared to the NC group. Administration of either VitD or RSV significantly elevated VE-cadherin content and upgraded Annexin A1 relative gene expression, compared to the diseased group. However, only RSV treatment significantly increased claudin 3 content compared to the T2DM group. The combination of both drugs displayed a more significant amelioration for both VE-cadherin and Annexin A1 as compared to monotherapy.

### 3.8. VitD, RSV, and their combination mitigated hippocampal neuroinflammation

**Fig 8:**
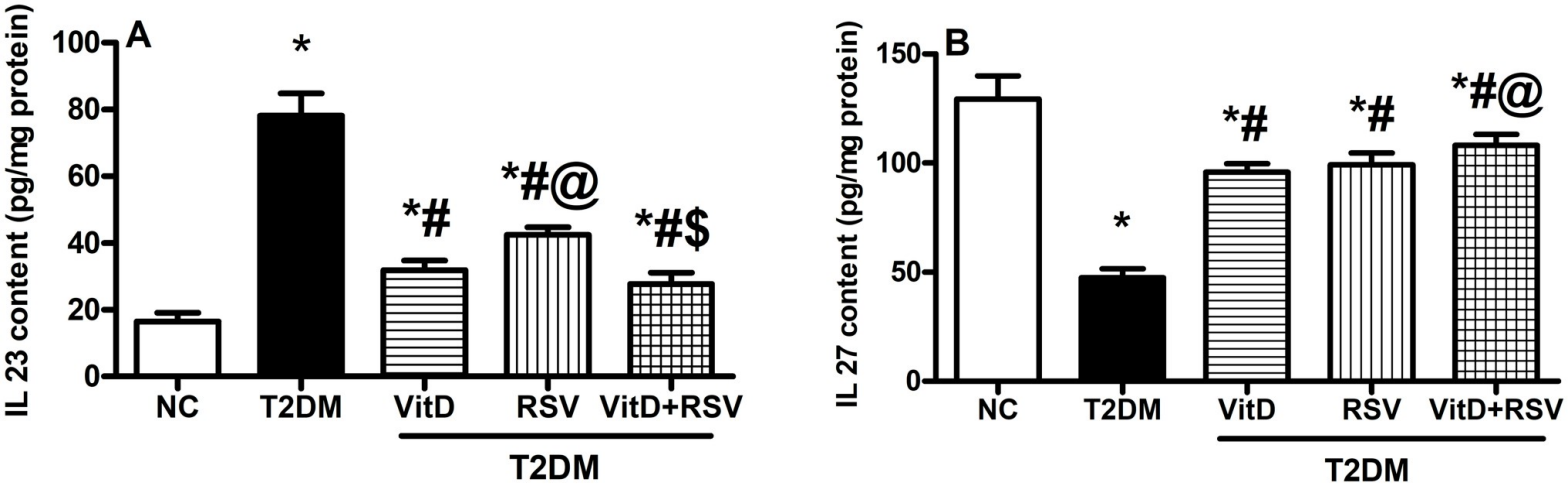
Effect of VitD and/or RSV on hippocampal T2DM-induced neuroinflammation. (A) IL-23; and (B) IL-27 contents. Data are expressed as mean ± SD. * vs control, ^#^ vs T2DM, ^@^ vs VitD, ^$^ vs RSV using one-way ANOVA followed by Tukey multiple comparison test at p<0.05. IL-23: interlukin-23, IL-27: interlukin-27, NC: normal-control, RSV: rosuvastatin, T2DM: type-II diabetes mellitus, VitD: vitamin D_3_.

Fig 8 showed that T2DM was associated with marked inflammatory events as evidenced by a 4.7-fold increment in the pro-inflammatory cytokine, IL-23 **(A)**, and a 63.3% reduction in the anti-inflammatory cytokine, IL-27 **(B)** as compared to the NC group. Treatment with VitD, RSV, or their combination showed significant anti-inflammatory effects through reducing IL-23 and elevating IL-27 levels, compared to the T2DM group.

### 3.9. VitD, RSV, and their combination hampered hippocampal Tau hyperphosphorylation and upregulated insulin and α7nACh receptors relative gene expression

**Fig 9:**
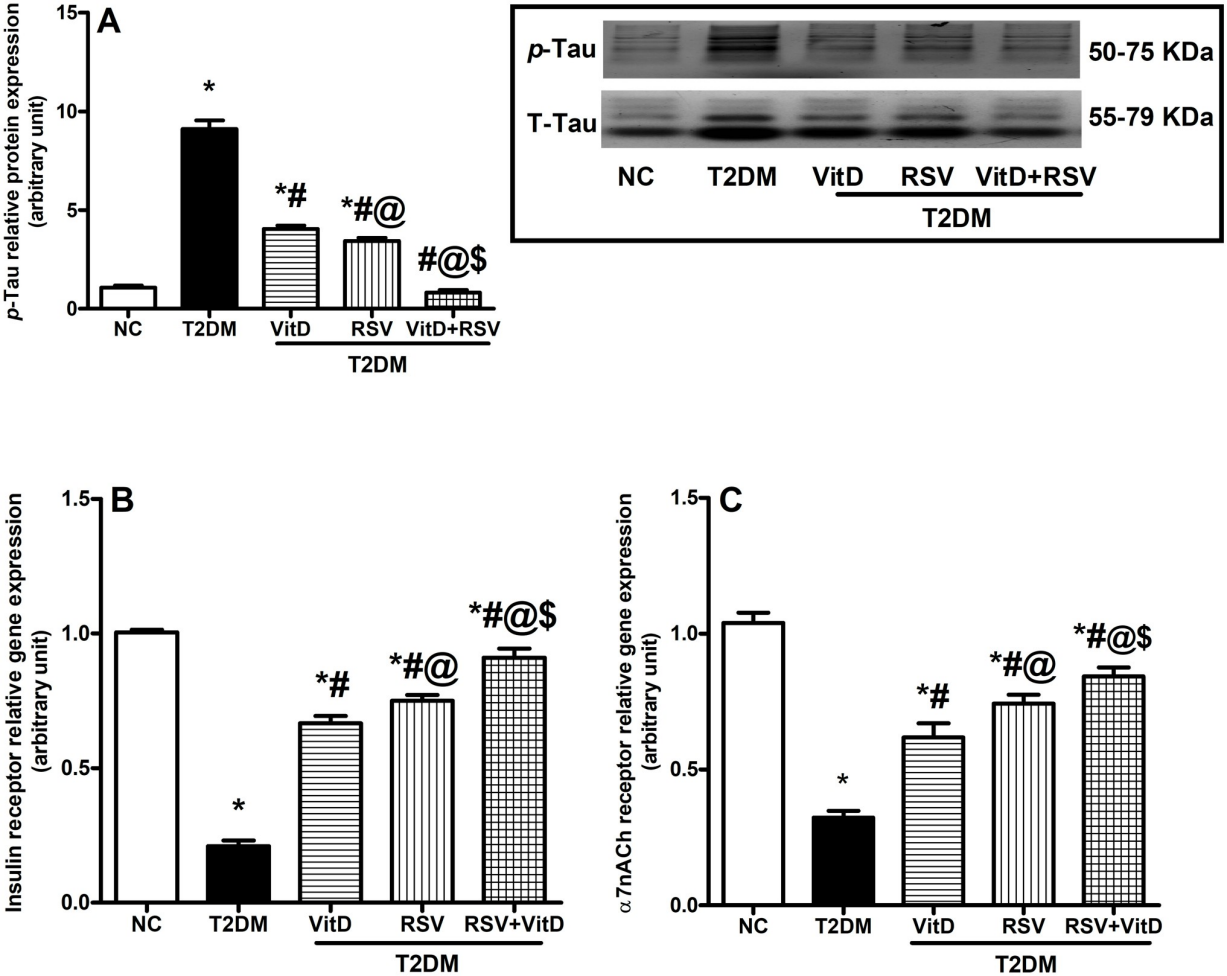
Effect of VitD and/or RSV on *p*-tau protein expression and gene expression of insulin and α7nACh receptors in the hippocampus. (A) *p*-tau protein, (B) insulin, and (C) α7nACh receptors. Data are expressed as mean ± SD. * vs control, ^#^ vs T2DM, ^@^ vs VitD, ^$^ vs RSV using one-way ANOVA followed by Tukey multiple comparison test at p<0.05. α7nACh: α7 nicotinic acetylcholine, NC: normal-control, RSV: rosuvastatin, T2DM: type-II diabetes mellitus, VitD: vitamin D_3_.

As shown in Fig 9, diabetic rats showed five times more phosphorylation of Tau protein **(A)**, accompanied by a 79% and 69% decline in the gene expression of insulin **(B)** and α7nACh **(C)** receptors as compared to the NC group. Treatment with either VitD or RSV alone lowered the level of *p*-tau and elevated the expression of both receptors, compared to T2DM. Again, the combined treatment was superior to either drug alone.

## 4. Discussion

Because of the epidemiological evidence for an increased risk of dementia and mild cognitive impairment in patients with diabetes, VitD and RSV were given to diabetic rats either alone or combined to investigate their protective potential in T2DM-induced memory deficits. This effect was partly attributed to (1) halting of T2DM-associated metabolic dysfunction, (2) modulation of the crosstalk between hippocampal insulin and noncanonical Wnt/β-catenin cassette, (3) stimulation of the canonical Wnt/β-catenin signaling pathway, (4) mitigation of neuroinflammation and preservation of BBB integrity, (5) improvement of memory and cognitive abilities, and 6) restoration of the hippocampal histological architecture.

Peripheral insulin resistance is accompanied by central manifestations like defective insulin signaling [45], neuroinflammation [46], brain abnormalities, as well as cognitive and memory deficits [47]. Remarkably, disrupted brain insulin pathways are accompanied by increased deposition of Aβ, Tau hyperphosphorylation, and the formation of neurofibrillary tangles (NFTs) [45]. In consistence, findings of the current work showed that maintaining rats on HFSD for eleven weeks with a single injection of STZ in the fourth week resulted in T2DM classical triad including hyperglycemia, insulin resistance, and dyslipidemia. These changes were accompanied by massive hippocampal injury as manifested by the profound neuronal loss, NFTs formation, neuroinflammation, and increased deposition of Aβ and Tau hyperphosphorylation with ensuing behavioral and memory deterioration as observed herein. T2DM is reported to induce impaired brain insulin functions through alteration of the PI3K/AKT/GSK-3β cascade [48]. Insulin and insulin receptors (IRs) are located in various brain regions [49]. They were found spread in the brain including the hippocampus [50] where it is anticipated to participate in cognitive function [51]. Further, amyloid-β peptides compete with insulin for binding to IR. This decreases the insulin binding affinity to IR and hence results in insulin resistance [52]. The dropped expression of hippocampal IRs in diabetic rats and its reversal by treatment, as reported in the current study, support the hypothesis that decreases in hippocampal IR activities contribute to behavioral deficits in type 2 diabetes [53].

The primary finding was that treatment of diabetic animals with VitD or RSV markedly improved T2DM-induced metabolic abnormalities in line with other reports [20, 21, 26, 28]. The beneficial effects of either VitD or RSV on the disrupted metabolic profile were paralleled by improved insulin sensitivity. VitD could increase insulin sensitivity either directly by stimulating the expression of insulin receptors [54, 55] and/or indirectly by lessening the effects of systemic inflammation in patients with T2DM. This could be achieved by protecting against β cell cytokine-induced apoptosis through modulating the expression and activity of cytokines and reducing chronic inflammation [56–59]. On the other hand, RSV increases insulin sensitivity in the whole body and peripheral tissues via improving cellular insulin signal transduction, in part, through increased activation of AKT [60]. It may also diminish the activity of inflammatory cascades including Jun N-terminal kinase and nuclear factor kappa-B pathways, that in turn improves insulin sensitivity since both are known to block insulin signaling through inhibition of IRS-1 [60].

Notably, the combined treatment with VitD and RSV provoked greater outcomes on the disrupted metabolic profile than either one alone. Interestingly, modulation of these metabolic abnormalities was reflected centrally and could be related to the ability of VitD and/ or RSV to improve defective insulin signaling by increasing the gene expression of hippocampal insulin receptors and protein expression of *p-*AKT and *p-*GSK-3β with reduced Tau hyperphosphorylation and Aβ deposition as shown in Figs 5 & 9 in parallel with other studies [61, 62]. VitD is involved in stimulating PI3K/AKT signaling, sensitizing the neuronal cells to downregulate the AD-like markers, particularly GSK-3β and Tau gene expression and amyloid-beta deposition [63, 64]. It seems that RSV reduces the risk of dementia due to its lipid-lowering effect. Lower cholesterol levels in the midlife help to reduce the risk of all types of dementia in late-life [65]. Furthermore, treatment with RSV ameliorated cognitive impairment by improved locomotor activity, reducing cholesterol deposition, acetylcholinesterase activity, and Aβ1–42 peptide aggregation [66]. VitD or RSV-induced molecular changes were corroborated with improved performance in the MWM and NOR tests and go in line with many investigators who reported their beneficial impacts on learning and memory [67–69].

Findings revealed that the protective effect of VitD in asthma [70], colon cancer [71, 72] and inflammatory bowel disease [73] is possibly through regulating the activity of Wnt/β-catenin signaling. VitD activates Wnt/β-catenin signaling pathway through modulating LDL Receptor Related Protein 5 (Lrp5) co-receptor (the main cofactor in Wnt/β-catenin pathway) [74]. Furthermore, VitD suppress (Dickkopf-1) DKK1 which is the main deactivator of the Wnt/β-catenin signaling pathway [75]. RSV, having pleiotropic effects, also modulates the Wnt/β-catenin signaling pathway [76] possibly through reducing the degradation of β-catenin and increasing its accumulation in the cells [77]. Indeed, administration of either drug significantly increased the hippocampal protein expression of the Wnt5a ligand, the main activator of the noncanonical Wnt pathway [78], with upregulation of RhoA and Rac1, phosphorylation of AKT, GSK-3β inhibition, Tau dephosphorylation and Aβ clearance [79]. Activation of the noncanonical Wnt pathway was reported to improve learning and memory deficits in various studies [18, 79]. Insulin resistance and hyperglycemia deactivate Wnt signaling and induce β-catenin degradation and nuclear dislocation [80]. Regarding VitD, the present findings showed for the first time that it resulted in activation of the noncanonical Wnt cascade and its downstream molecules RhoA and Rac1.

As for the canonical Wnt/β-catenin cassette, it was activated following the administration of VitD, RSV, or their combination. Inhibition of canonical Wnt/β-catenin pathway leads to enhanced phosphorylation of β-catenin by GSK-3β that mediated its ubiquitination and proteasomal degradation as observed herein [17]. However, administration of VitD and/or RSV modulated the canonical Wnt/β-catenin trajectory as evidenced by the increased protein expression of Wnt5a and *p*S675 β-catenin, as well as reduced ApoE-4 hippocampal levels. Hence, the enhanced Wnt/β-catenin signaling with subsequent stimulation of its nuclear targets could pin down a key mechanism by which VitD or RSV may improve T2DM provoked hippocampal injury and associated cognitive and memory impairment.

Among activated Wnt/ β-catenin transcriptional targets are genes encoding for tight junction proteins Annexin A1 [81] and claudin 3 [82], as well as adherens junction proteins namely VE-cadherin [83]. The present study demonstrated that administration of VitD and/or RSV markedly upregulated the protein expression of Annexin A1 and claudin 3 paralleled by a pronounced reduction in neuronal loss, NFTs, and Aβ deposition. Regarding VE-cadherin, its downregulation triggers BBB leakage, which is involved in CNS pathologies like AD [84] as observed herein. Notably, administration of VitD and/or RSV to T2DM rats upsurged the hippocampal levels of VE-cadherin in line with previous studies [85–87].

Another important downstream target for Wnt /β-catenin signaling is α7 nicotinic acetylcholine receptor (α7nAChR) [88] whose downregulation in the hippocampus and cortex correlates with Aβ-induced neurotoxicity and cognitive dysfunction [89]. The present findings demonstrated that VitD administration upregulated the gene expression of α7nAChR, an effect that could be ascribed to its ability to turn on the Wnt/β-catenin hub. Similarly, RSV upsurged the gene expression of α7nAChR which is quite consistent with *Chen et al*. [90]. Remarkably, the administration of both agents produced a greater effect than either one alone, suggesting the benefits of the combination treatment. The upregulated gene expression of α7nAChR goes in line with many authors [91–93].

The findings of the current work showed that VitD or RSV increased the anti-inflammatory, IL 27 and decreased the proinflammatory, IL 23 cytokines’ levels. This was further augmented by the co-administration of both drugs. Regulating the expression of these pivotal cytokines is one of the Wnt/β-catenin downstream signaling [94] roles in maintaining the balance between anti-inflammatory and proinflammatory cytokines, preserving the BBB integrity, and improving learning and memory deficits [95]. The ability of VitD and/or RSV to suppress neuroinflammation is either related to their direct anti-inflammatory effects or to their aptitude to modulate the crosstalk between impaired insulin/AKT/GSK-3β and canonical/ noncanonical Wnt/β-catenin pathways. Again, such molecular effects were mirrored histopathologically and behaviorally.

## 5. Conclusion

Taken altogether, the current study accentuated the neuroprotective potential of VitD and/or RSV in ameliorating T2DM-induced hippocampal insult and accompanied behavioral alterations. These protective effects include modulation of the intersection between insulin/AKT/GSK-3β and canonical/non-canonical Wnt/β-catenin trajectories, as well as mitigation of neuroinflammation with subsequent improvement in memory and cognitive defects, as well as restoration of the hippocampal histological profile. The present work provides novel incentives for the use of RSV and/or VitD to slow down T2DM-induced neuronal injury. Further studies are warranted to determine their benefits in clinical practice.

## 6. Limitation of the study

It is important to remember that even though insulin resistance is the core pathology of diabetes, there are several metabolic consequences that should also be taken into consideration. In addition, effects of the drugs used on the signaling pathways were studied in the whole hippocampal region; further studies may be needed to determine which sub-regions are responsible for the observed outcomes. Furthermore, apart from the studied pathways, more cascades need to be assessed to elucidate other mechanisms by which the examined agents can act.

## Supporting information

S1 File (PDF)
