# Supplementary figures and images for: Vitamin D and rosuvastatin alleviate type-II diabetes-induced cognitive dysfunction by modulating neuroinflammation and canonical/noncanonical Wnt/β-catenin signaling
Source: PLoS One. 2022 Nov 14;17(11):e0277457. doi: 10.1371/journal.pone.0277457 (PMC9662739; doi:10.1371/journal.pone.0277457)

# AKT

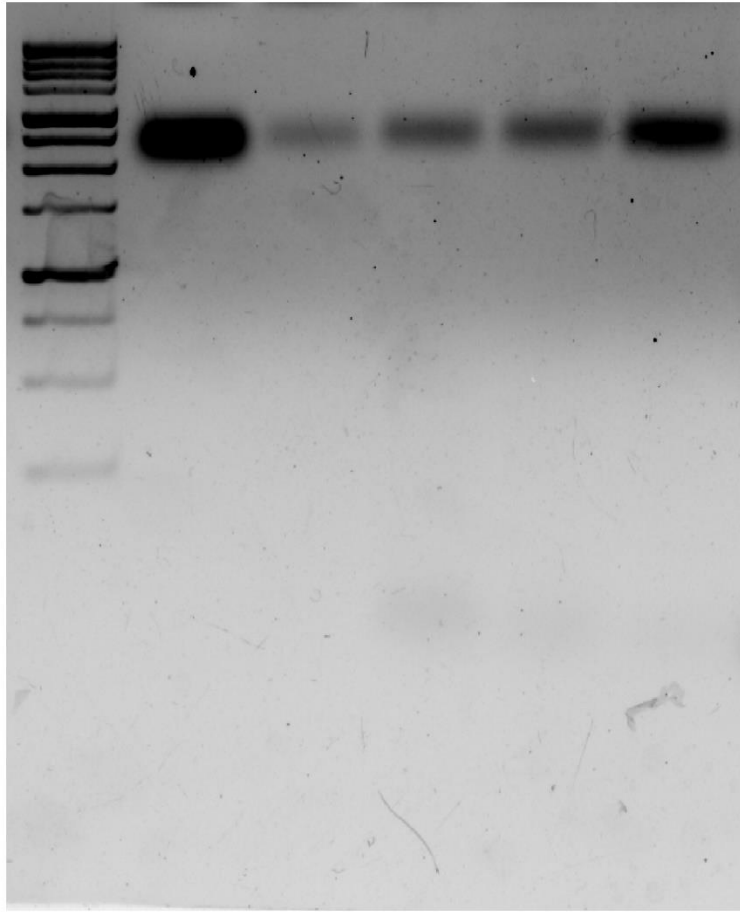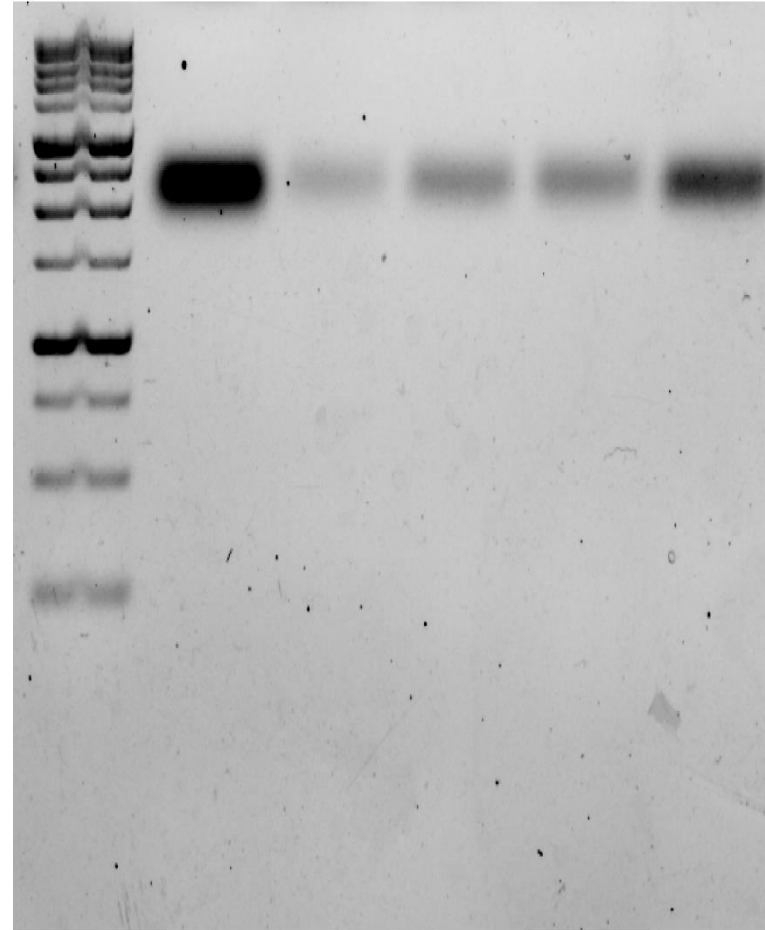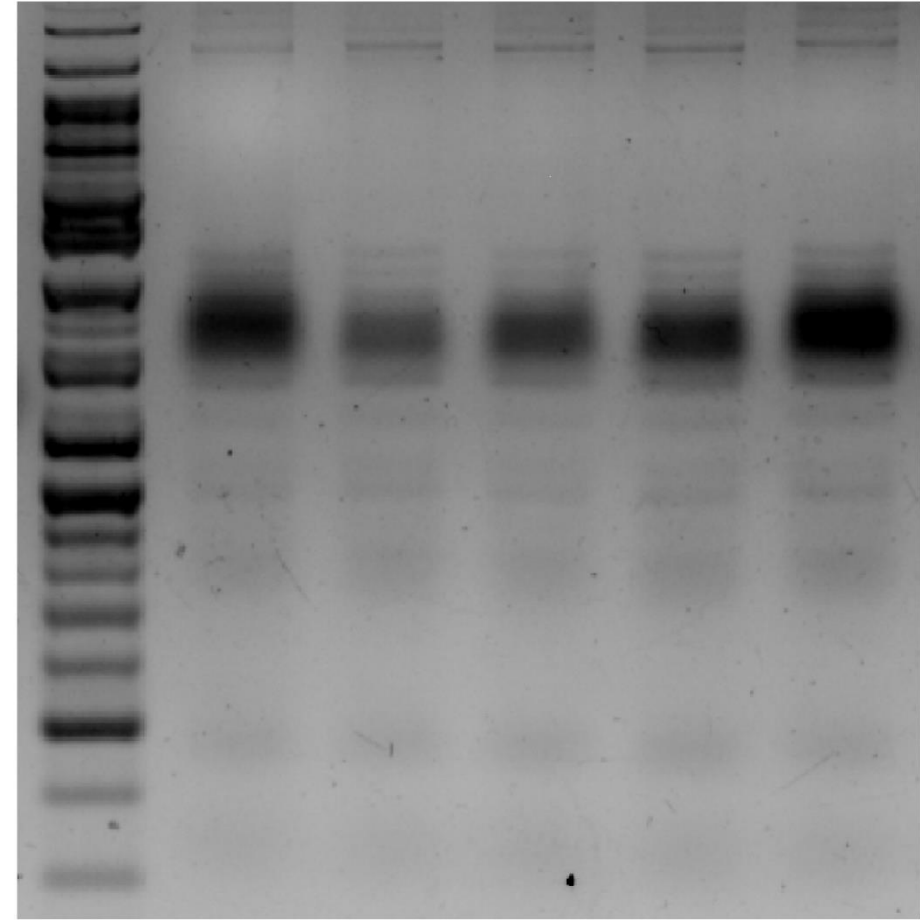

# GSK-3 $\beta$

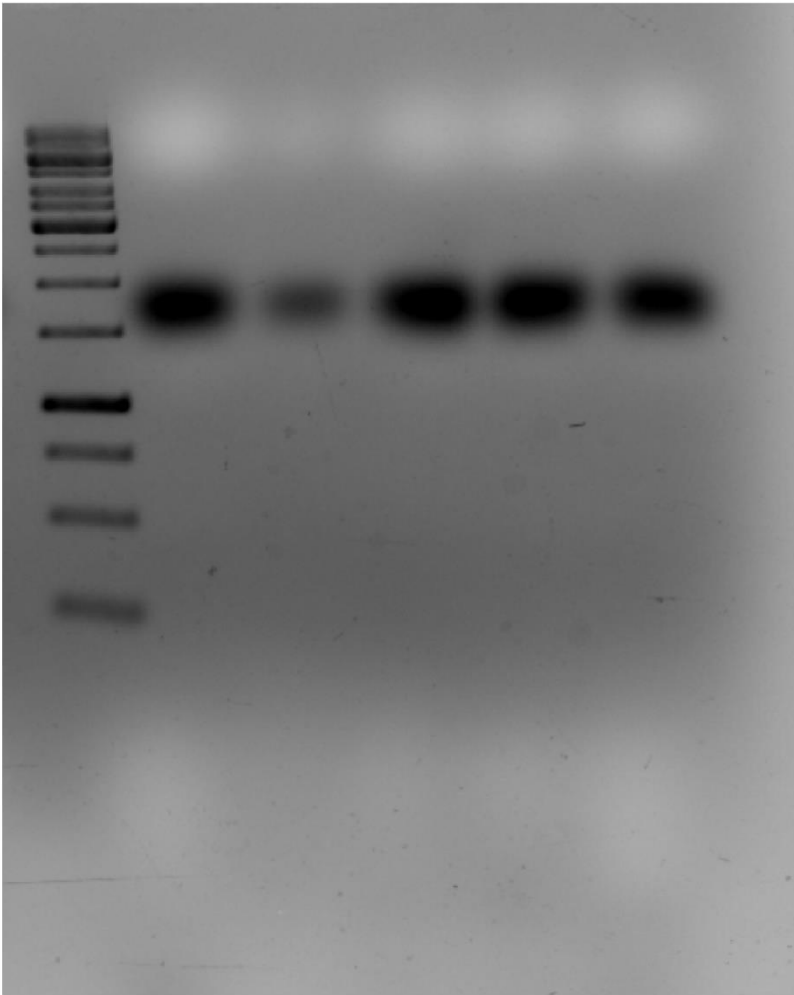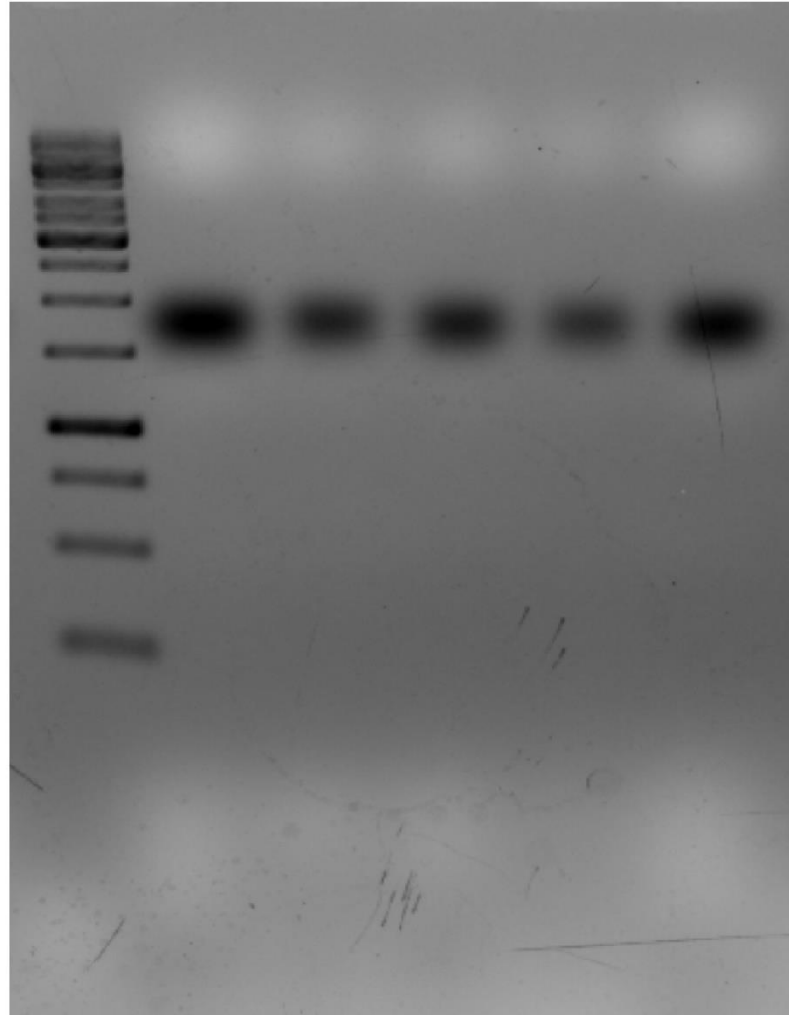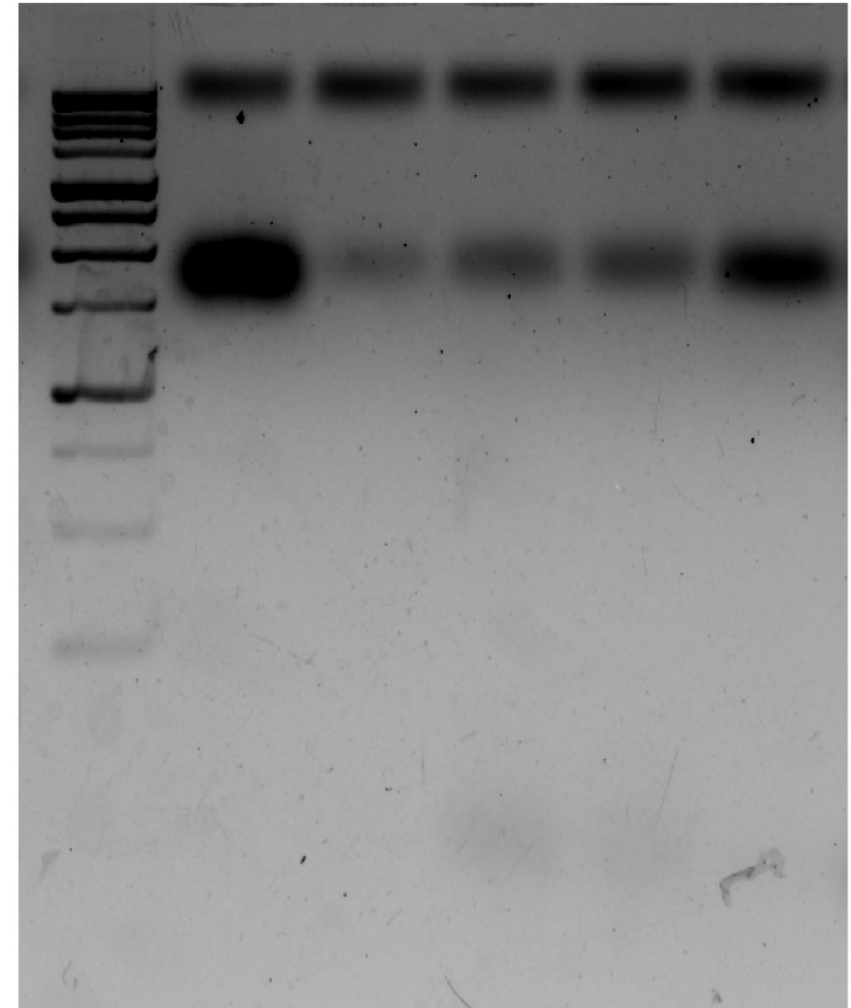

# Rac-1

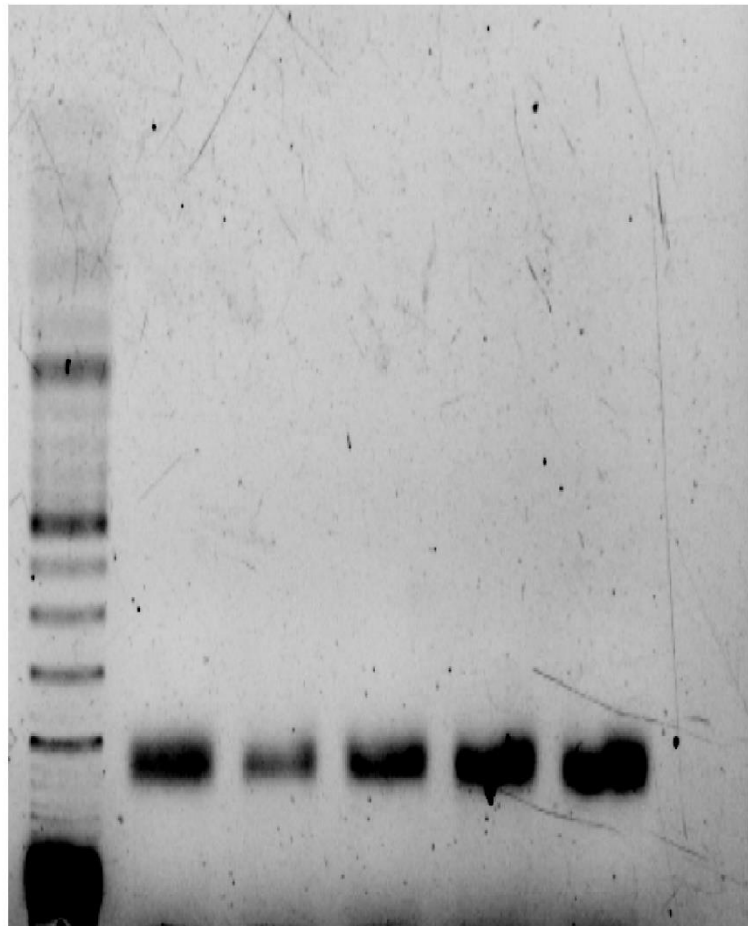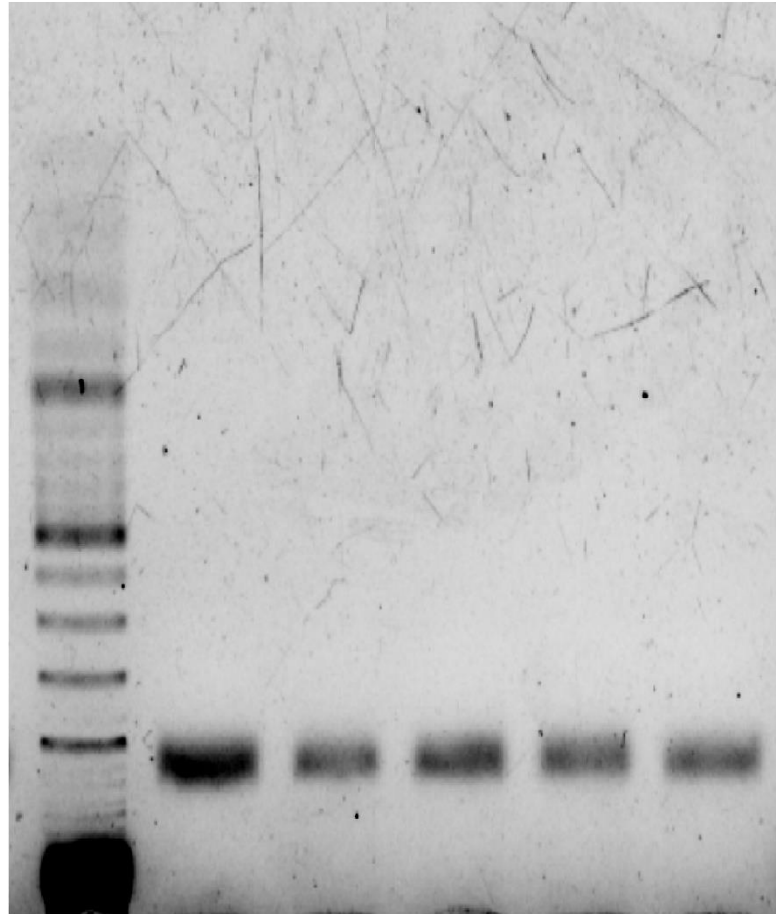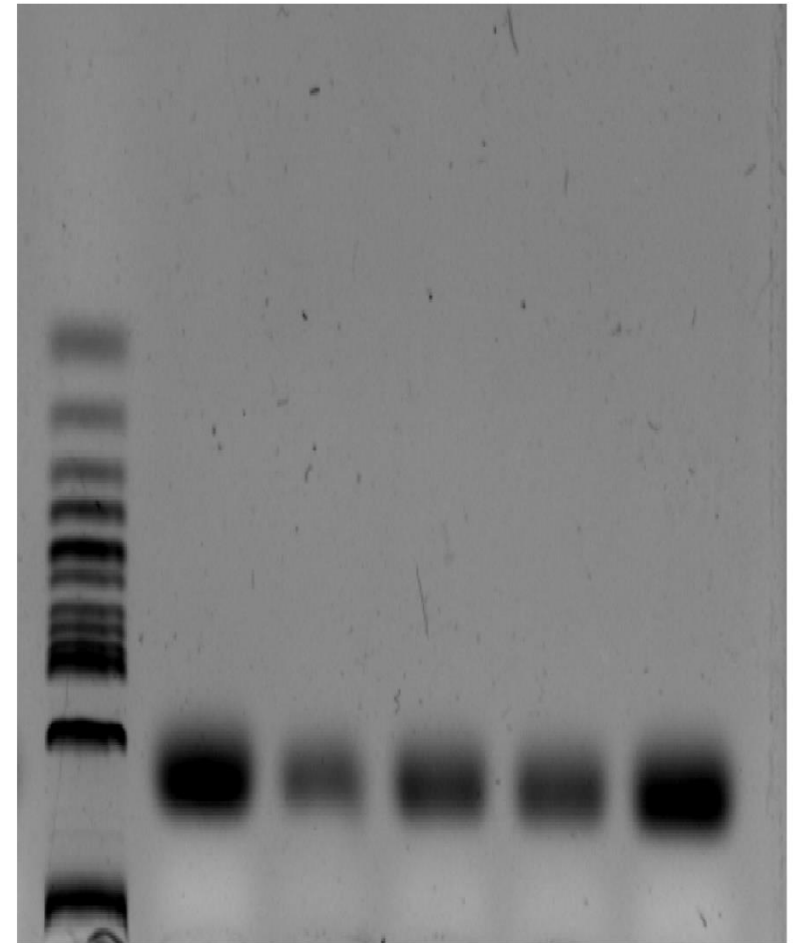

# RohA

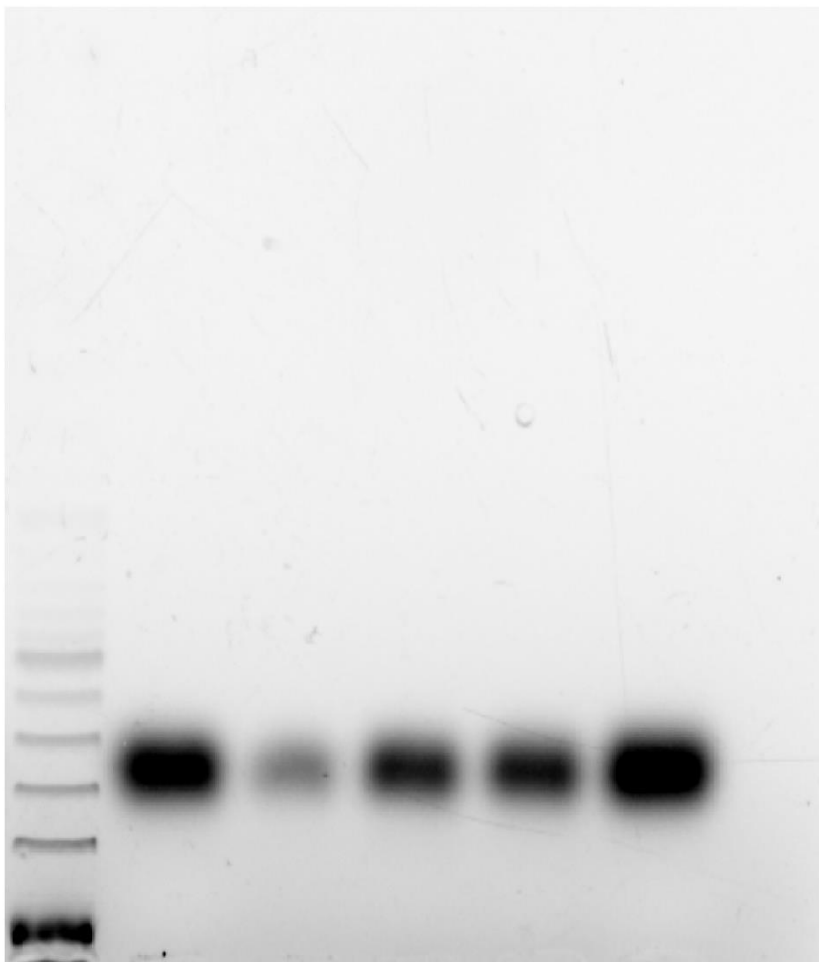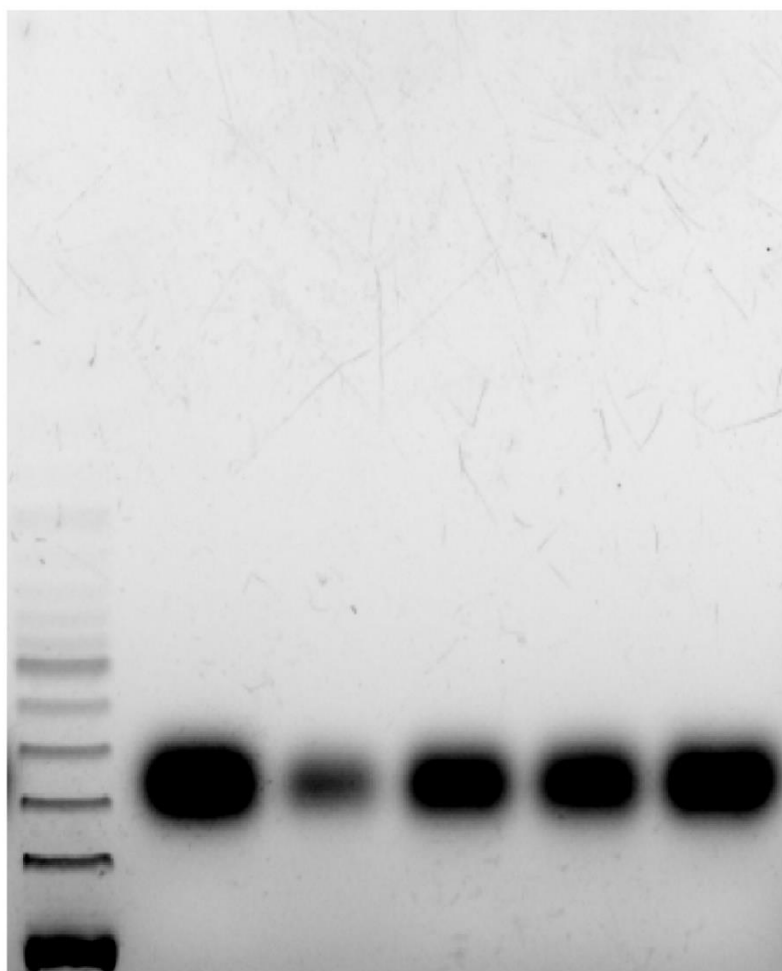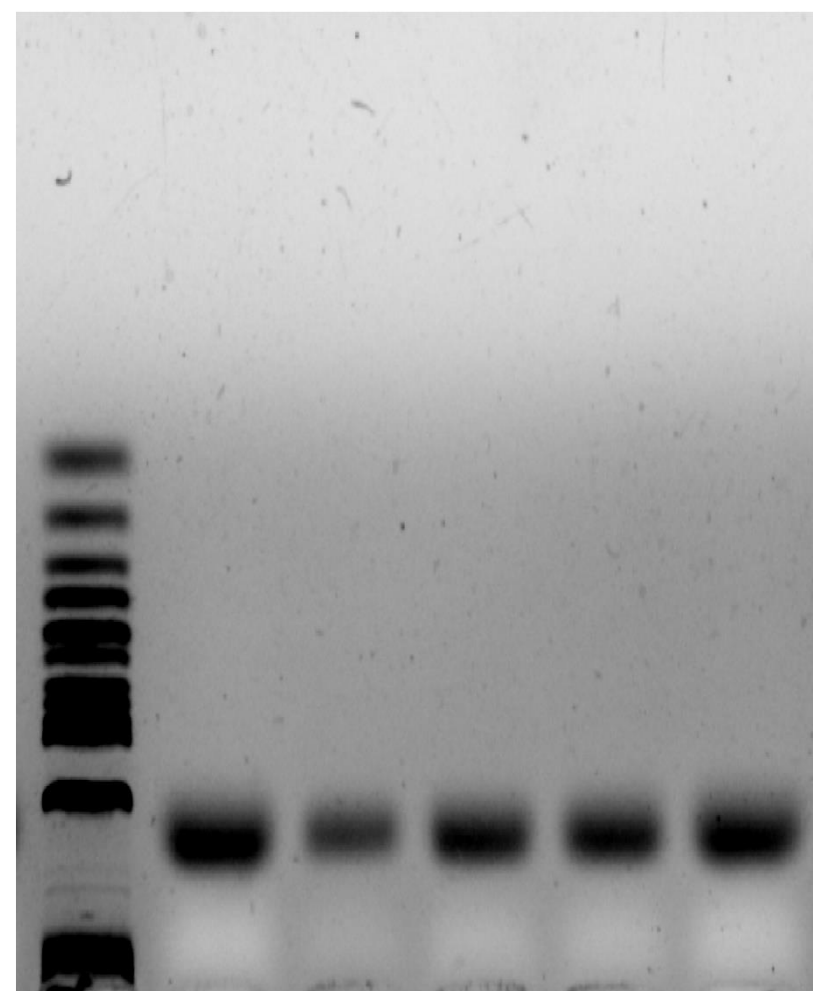

# $\beta$ -catenin 37

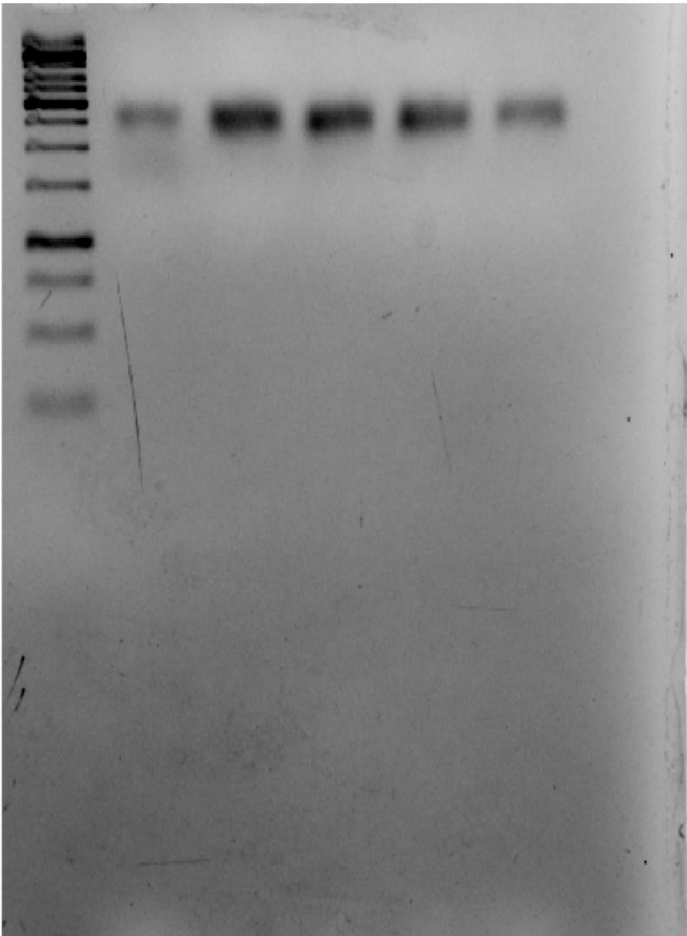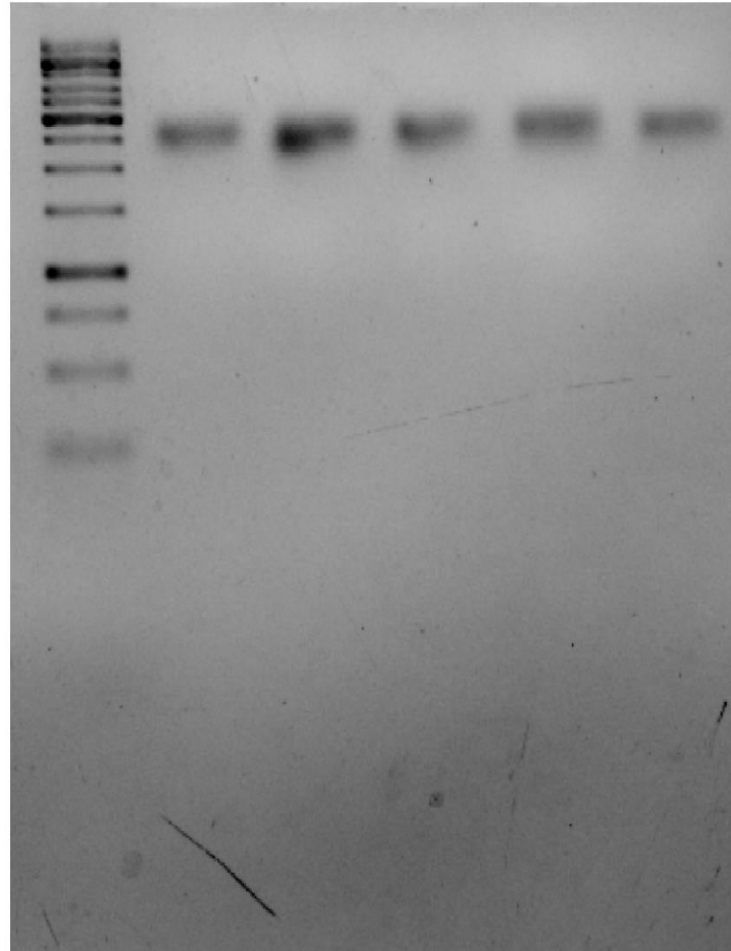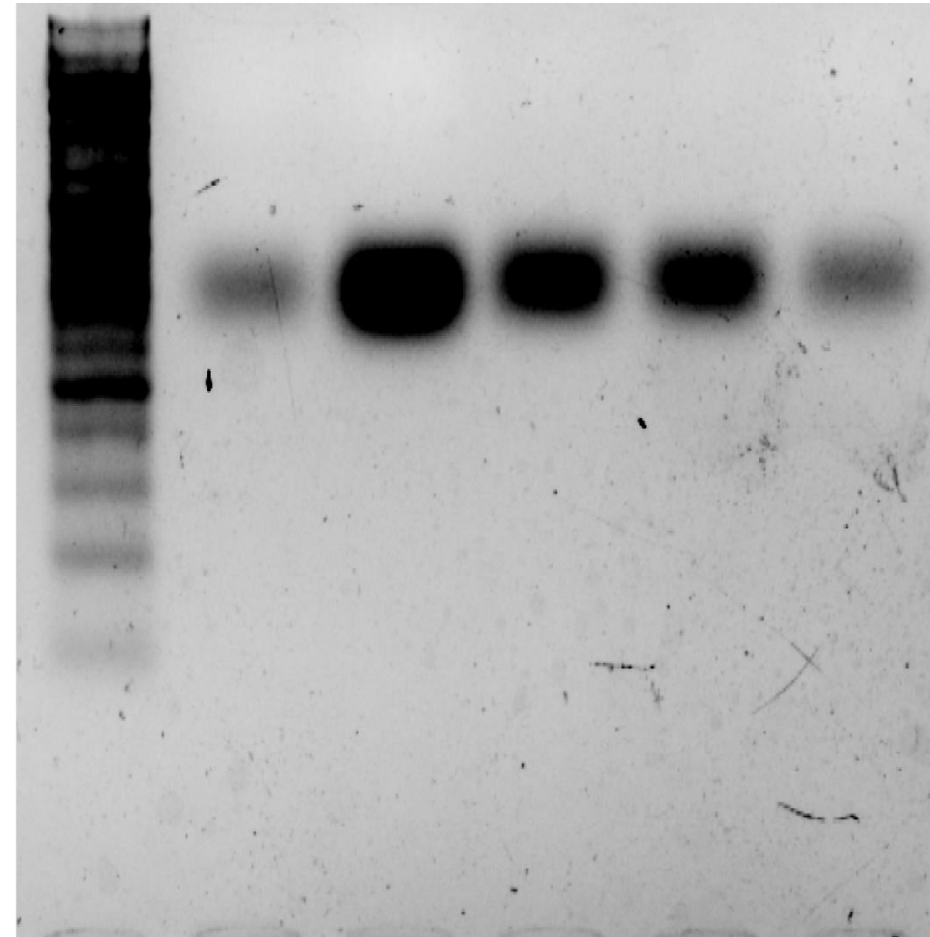

# $\beta$ -catenin 675

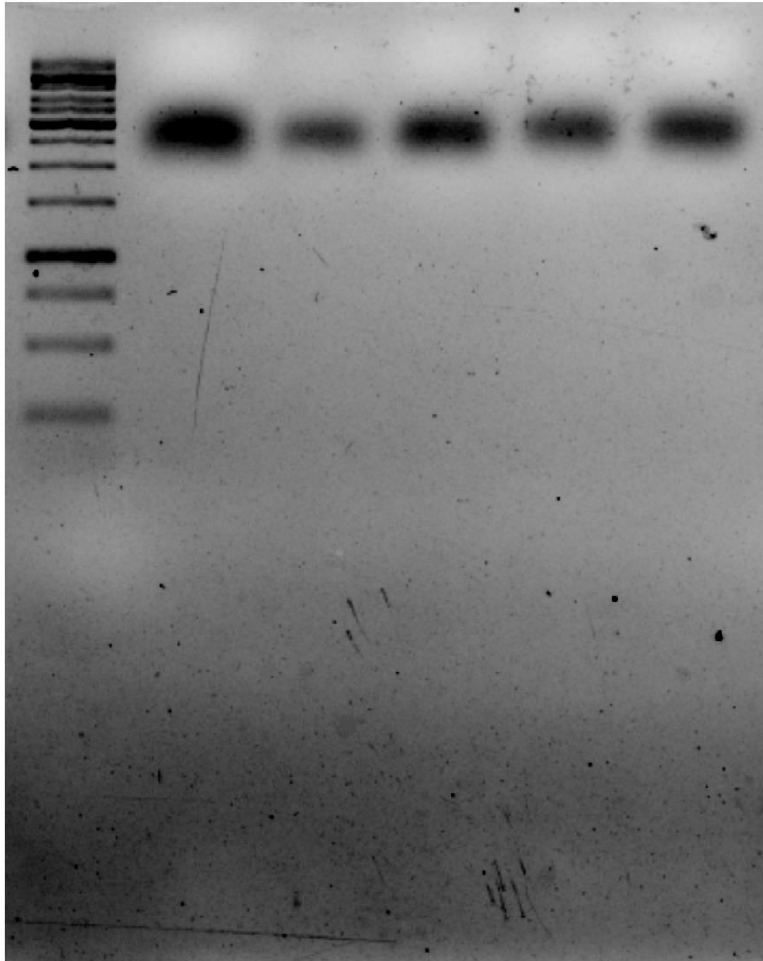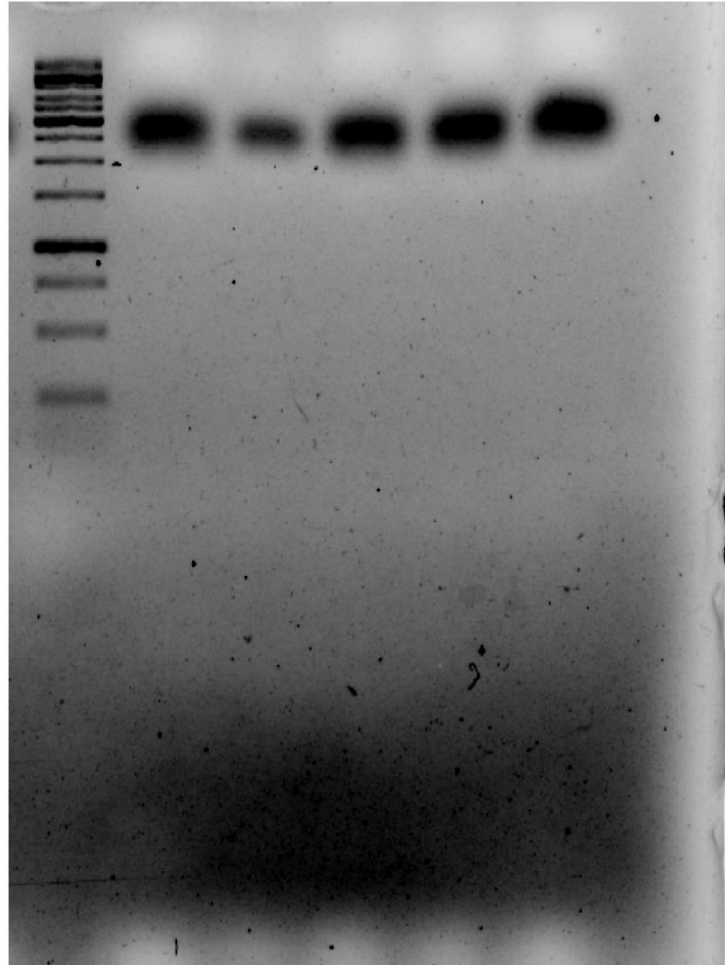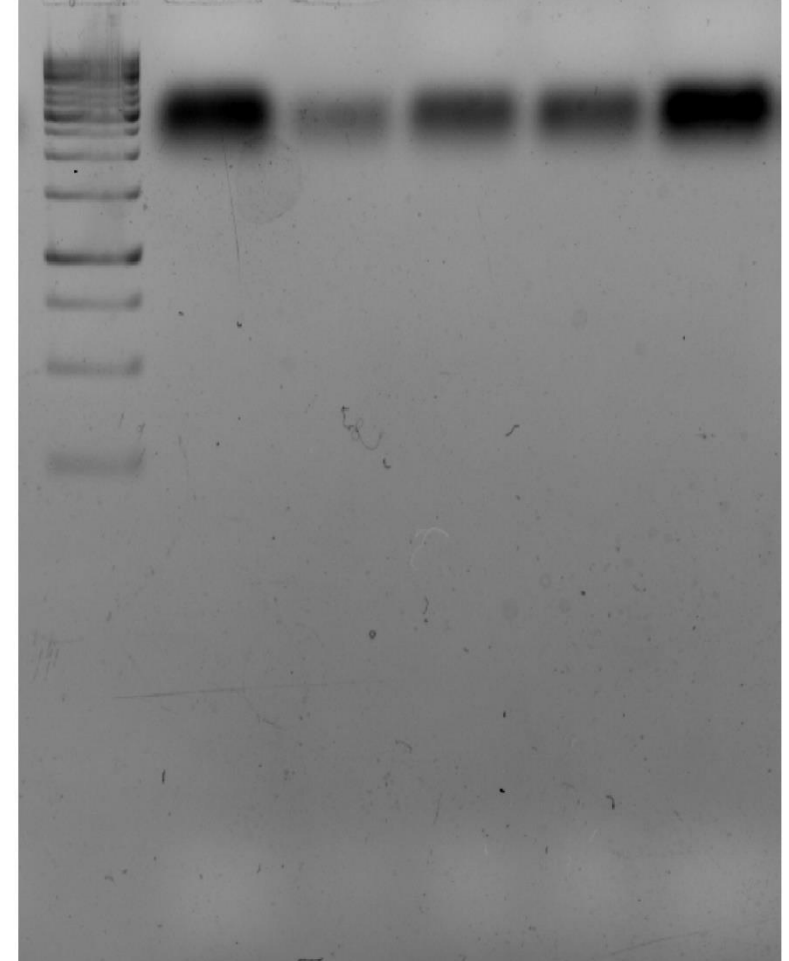

# $\beta$ -actin

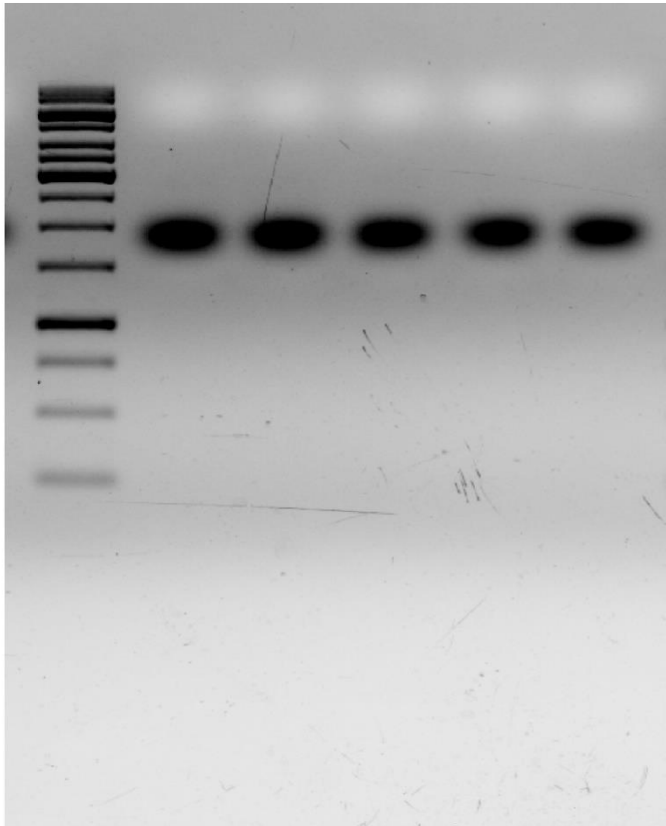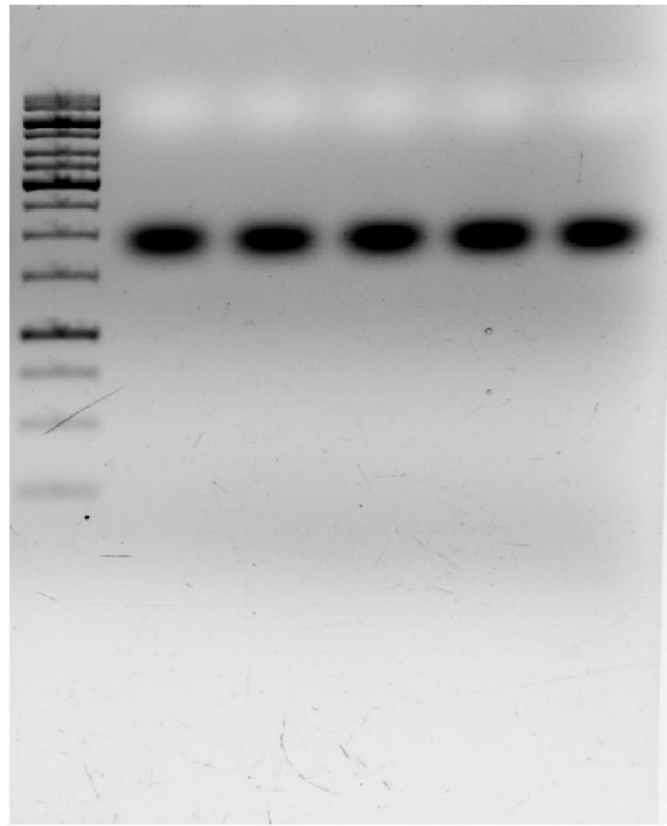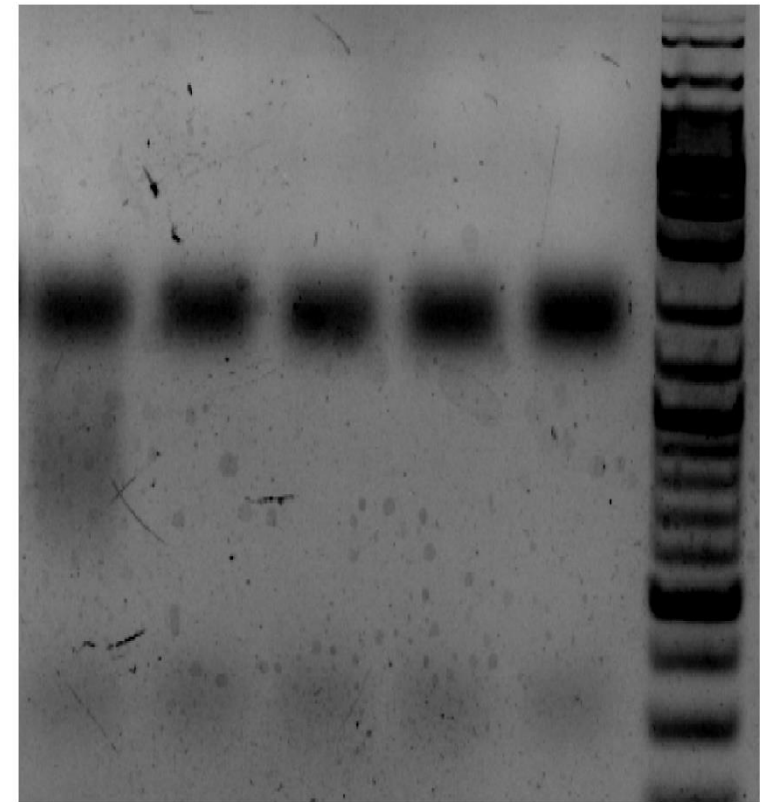

# Wnt-5a

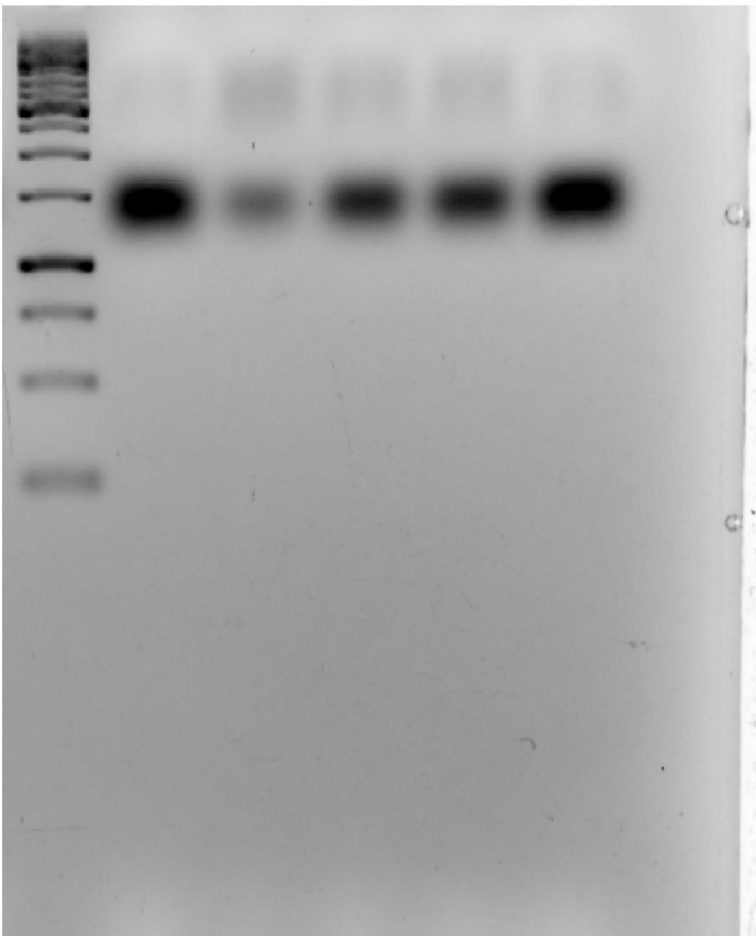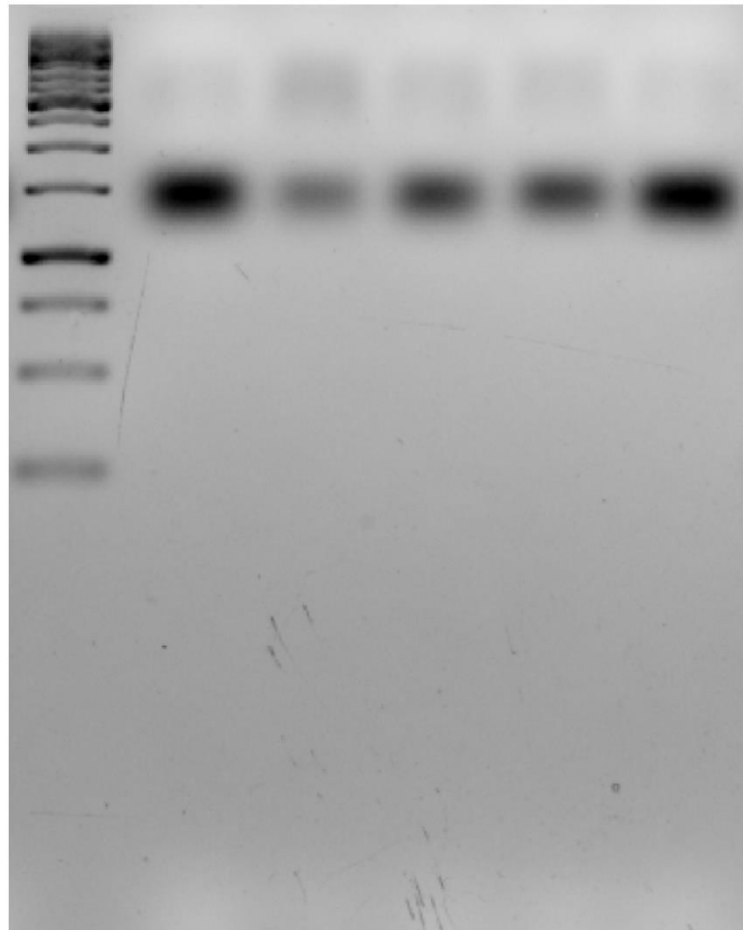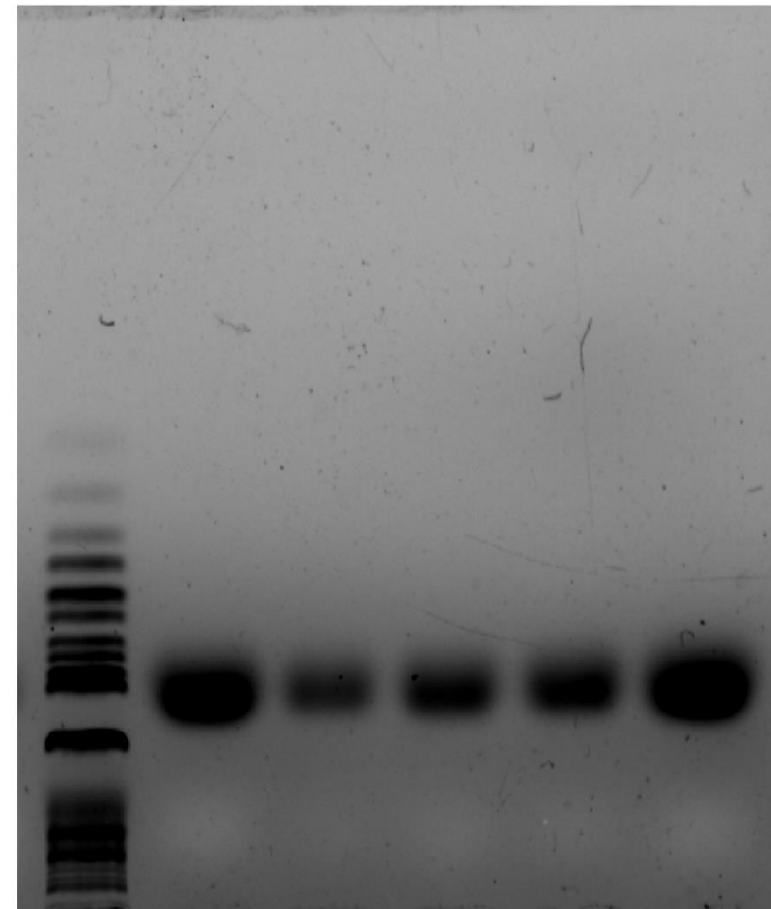

Supplement: S1 File — (PDF) [file pone.0277457.s001.pdf]
